# *Campylobacter jejuni* Demonstrates Conserved Proteomic and Transcriptomic Responses When Co-cultured With Human INT 407 and Caco-2 Epithelial Cells

**DOI:** 10.3389/fmicb.2019.00755

**Published:** 2019-04-11

**Authors:** Nicholas M. Negretti, Geremy Clair, Prabhat K. Talukdar, Christopher R. Gourley, Steven Huynh, Joshua N. Adkins, Craig T. Parker, Colby M. Corneau, Michael E. Konkel

**Affiliations:** ^1^School of Molecular Biosciences, College of Veterinary Medicine, Washington State University, Pullman, WA, United States; ^2^Integrative Omics, Pacific Northwest National Laboratory, Richland, WA, United States; ^3^Produce Safety and Microbiology, United States Department of Agriculture-Agricultural Research Service, Albany, CA, United States

**Keywords:** protein synthetic response, gene expression, bacteria–host cell interactions, host cell invasion, proteomics

## Abstract

Major foodborne bacterial pathogens, such as *Campylobacter jejuni*, have devised complex strategies to establish and foster intestinal infections. For more than two decades, researchers have used immortalized cell lines derived from human intestinal tissue to dissect *C. jejuni*-host cell interactions. Known from these studies is that *C. jejuni* virulence is multifactorial, requiring a coordinated response to produce virulence factors that facilitate host cell interactions. This study was initiated to identify *C. jejuni* proteins that contribute to adaptation to the host cell environment and cellular invasion. We demonstrated that *C. jejuni* responds to INT 407 and Caco-2 cells in a similar fashion at the cellular and molecular levels. Active protein synthesis was found to be required for *C. jejuni* to maximally invade these host cells. Proteomic and transcriptomic approaches were then used to define the protein and gene expression profiles of *C. jejuni* co-cultured with cells. By focusing on those genes showing increased expression by *C. jejuni* when co-cultured with epithelial cells, we discovered that *C. jejuni* quickly adapts to co-culture with epithelial cells by synthesizing gene products that enable it to acquire specific amino acids for growth, scavenge for inorganic molecules including iron, resist reactive oxygen/nitrogen species, and promote host cell interactions. Based on these findings, we selected a subset of the genes involved in chemotaxis and the regulation of flagellar assembly and generated *C. jejuni* deletion mutants for phenotypic analysis. Binding and internalization assays revealed significant differences in the interaction of *C. jejuni* chemotaxis and flagellar regulatory mutants. The identification of genes involved in *C. jejuni* adaptation to culture with host cells provides new insights into the infection process.

## Introduction

Pathogens must navigate a gauntlet of hostile host environments and defenses to eventually cause disease in the human intestine. The constantly changing host conditions encountered by bacteria when they are ingested and passed through the alimentary canal provide cues to alter their behavior. Pathogens employ strategies ranging from alteration of metabolic activity to differential expression of genes and gene products. Deciphering the molecular behavior of microbes within a host is key to understanding how disease progression occurs and informs strategies that could ultimately mitigate disease.

*Campylobacter jejuni* is one of the most common bacterial causes of foodborne illness worldwide and is estimated to be responsible for between 400 and 500 million cases of gastroenteritis each year (Ruiz-Palacios, 2007). Early in infection, *C. jejuni* colonize and invade the intestinal epithelial cells, resulting in symptoms ranging from fever and abdominal cramping to diarrhea containing blood and immune cells. Disease symptoms are more severe in populations such as the very young, elderly, and chronically ill. *C. jejuni* virulence is multifactorial, requiring motility, translocation of the intestinal barrier, host (target) cell adherence, host cell invasion, alteration of host cell signaling pathways, induction of host cell death, evasion of host immune defenses, iron acquisition, and drug/detergent resistance (Johanesen and Dwinell, 2006; Eucker and Konkel, 2012; Neal-McKinney and Konkel, 2012; Backert and Hofreuter, 2013). This list is not comprehensive, but rather, illustrates that *C. jejuni* disease occurs in a susceptible host from a combination of virulence attributes working in concert.

*In vitro* tissue culture models have been used extensively to assess the virulence potential of *C. jejuni* isolates recovered from both clinical and environmental sources. These studies have led to the identification of proteins that facilitate the binding and invasion of *C. jejuni* to host cells. Many of the proteins that promote the binding of *C. jejuni* to host cells, including CadF and FlpA, are synthesized constitutively (Konkel and Cieplak, 1992; Konkel et al., 2007). In contrast, cellular invasion requires *de novo* protein synthesis that occurs in response to a stimulatory signal (i.e., contact with host cells) (Konkel and Cieplak, 1992; Neal-McKinney and Konkel, 2012). Moreover, metabolic labeling and immunoblot analyses have revealed that co-culture of *C. jejuni* with human INT 407 cells results in changes in the synthesis of proteins compared with the proteins synthesized by *C. jejuni* cultured in the absence of the epithelial cells (Konkel and Cieplak, 1992; Konkel et al., 1993; Eucker et al., 2014). In a separate study, Panigrahi et al. (1992) found that *C. jejuni* synthesizes proteins in a rabbit ileal loop that are not expressed under standard laboratory culture conditions. A subset of the newly synthesized proteins reacted with convalescent sera from *C. jejuni*-infected individuals. Relevant to this study, *C. jejuni* also synthesizes a similar subset of unique proteins when co-cultured with human INT 407 epithelial cells (Konkel and Cieplak, 1992; Konkel et al., 1993). Despite these previous observations, a global account of the overall changes in gene expression and protein synthesis during *C. jejuni* co-culture with host cells is lacking.

The purpose of this study was to gain a better understanding of the response of *C. jejuni* to co-culture with human epithelial cells. By utilizing both proteomic and transcriptomic analyses of *C. jejuni* strain 81-176 co-cultured with human INT 407 cells and human colonic Caco-2 cells, we identified genes that encode products that promote the survival and interaction of *C. jejuni* with host cells. To assess the relevance of the findings, deletion mutants were created for genes involved in chemotaxis and flagellar assembly and tested for the contribution in cellular adherence and invasion. Our study has revealed that *C. jejuni* flagellar regulatory and structural mutants display a gross difference in host cell interactions when compared to chemotaxis mutants. The findings present a refined view of *C. jejuni* virulence factors that promote cell interactions.

## Materials and Methods

### Bacterial Strains

*Campylobacter jejuni* wild-type strains 81–176 and F38011 were cultured on Mueller-Hinton agar (Hardy Diagnostics, Santa Maria, CA, United States) containing 5% citrated bovine blood (MHB agar), or in Mueller-Hinton broth (MH broth) on an orbital shaker at 225 rpm under microaerobic (5% O_2_, 10% CO_2_, 85% N_2_) conditions at 37°C in a Napco 8000WJ incubator (Thermo Fisher, Waltham, MA, United States), with routine subculture on MHB agar every 24–48 h. Where applicable, MHB agar and MH broth were supplemented with chloramphenicol (8 μg/mL) or hygromycin B (250 μg/mL).

### Host Epithelial Cell Lines

INT 407 (ATCC CCL-6) and Caco-2 (ATCC HTB-37) cells were cultured in Minimal Essential Media (MEM; Gibco, Grand Island, NY, United States) supplemented with 10% fetal bovine serum (FBS; GE Healthcare Life Sciences, HyClone Characterized Fetal Bovine Serum US Origin cat. # SH30071) and 1 mM sodium pyruvate (Corning Inc., Manassas, VA, United States) at 37°C with 5% CO_2_. Infection and metabolic labeling studies were done in a T-75 flask with either INT 407 or Caco-2 cells that were seeded at a density of 5 × 10^6^ cells/flask (∼70% confluence) and grown for 24 h.

### Metabolic Labeling Assays

*Campylobacter jejuni* were harvested from MHB agar plates in phosphate buffered saline (PBS). Bacteria were washed twice in Eagle’s Minimal Essential Medium (EMEM) lacking L-methionine (labeling medium; MP Biomedicals LLC cat. # 1641454 supplemented with 0.1 mM L-cysteine and 2 mM L-glutamine) and suspended in medium to an optical density (OD_540_) of 0.3 (∼1.5 × 10^9^ cfu/mL). Metabolic labeling experiments were performed in 3 mL of labeling medium containing 75 μCi [^35^S]-methionine (PerkinElmer, Boston, MA, United States) and 1% FBS. The FBS used in these experiments was albumin-depleted and dialyzed, as outlined by the manufacturer (Thermo Scientific, SwellGel Blue Albumin Removal Kit, Product # 89845). Emetine hydrochloride (2.5 μg/mL, Sigma-Aldrich, St. Louis, MO, United States) was added to the labeling medium 30 min prior to the addition of [^35^S]-methionine in order to inhibit eukaryotic protein synthesis. Assays were also conducted with INT 407 cells that were fixed with 4.0% paraformaldehyde (Electron Microscopy Sciences, Hatfield, PA, United States) in PBS (pH 6.9) for 15 min at room temperature. The paraformaldehyde–fixed INT 407 cells were washed 5 times with labeling medium before the addition of the bacteria. Following metabolic labeling, the bacterial pellets were washed with PBS and the amount of [^35^S]-methionine incorporation into proteins was determined by trichloroacetic acid (TCA; JT Baker Chemical Company, Center Valley, PA, United States) precipitation.

### Adherence and Internalization Assays

Binding and internalization (gentamicin protection) assays were performed with INT 407 and Caco-2 cells as described previously (Christensen et al., 2009; Eucker and Konkel, 2012). Briefly, *C. jejuni* were suspended to an OD_540_ of 0.03 and treated with medium alone or chloramphenicol at the indicated concentrations for 30 min prior to the addition of the *C. jejuni* to INT 407 cells. The epithelial cells were incubated at 37°C for the indicated times with chloramphenicol. Following an incubation period to allow the bacteria to invade the cells, the epithelial cell monolayers were rinsed with PBS and incubated for an additional 3 h with 250 μg/mL gentamicin to kill the extracellular bacteria. The epithelial cells were lysed with a solution of 0.1% Triton X-100 in PBS. All assays were performed at a multiplicity of infection (MOI) ranging between 50 and 500, and repeated a minimum of three times to ensure reproducibility. The reported values represent the mean counts ± standard deviations derived from triplicate wells. Where indicated, chloramphenicol (GoldBio, St. Louis, MO, United States) was added to inhibit bacterial protein synthesis (the specific concentrations used are indicated in the text).

### RNA-Seq Sample Preparation

*Campylobacter jejuni* were inoculated at an OD_540_ of 0.05 and grown for 18–20 h in MH broth. The *C. jejuni* was then adjusted to an OD_540_ of 0.3 in MEM supplemented with 1% FBS. INT 407 or Caco-2 cells were first rinsed with MEM 1% FBS, then 10 mL of the *C. jejuni* suspension was introduced and the host cells were incubated for 2.5 or 4 h at 37°C with 5% CO_2_. The supernatant was then collected and bacteria were recovered by centrifugation. For RNA analysis, bacteria were collected in 1/10 volume of ice cold stop solution (5% phenol, 95% ethanol), flash frozen in liquid nitrogen within 5 min of sample collection, and stored at -80°C until RNA extraction.

### RNA Extraction, rRNA Depletion, and Sequencing

Total RNA was isolated and genomic DNA removed using the Ambion Ribopure Bacteria kit after thawing bacterial pellets in the included RNAwiz solution following manufacturers protocols (Thermo Fisher, Waltham, MA, United States). Ribosomal RNA was depleted as described elsewhere (Rey et al., 2010). Briefly, 3′ biotinylated oligos with a tetraethylene glycol spacer were designed to hybridize to the *C. jejuni* 16S and 23S rRNAs. Two micrograms of each RNA sample was suspended in TES buffer (10 mM Tris, 1 mM EDTA, 1 M NaCl, pH 8.0) and mixed with 50 pmol of oligos. The samples were then incubated at 70°C for 15 min followed by 37°C for 15 min. TES equilibrated streptavidin coated agarose beads (GoldBio, Olivette, MO, United States) were used to capture depletion oligos and bound rRNA. rRNA depletion was assessed with an Advanced Analytical Fragment Analyzer (Ankeny, IA, United States). Illumina MiSeq libraries were prepared using the KAPA stranded RNAseq kit (Kapa Biosystems, Wilmington, MA, United States), following the manufacturer’s instructions except for the following changes: 159–400 ng RNA was sheared for 6 min at 85°C. Standard desalted TruSeq LT primers (Integrated DNA Technologies, Coralville, IA, United States) were used at 50–100 nM final concentration based on starting RNA amount. The PCR step was reduced to 6 cycles. Libraries were quantified using the KAPA Library Quantification Kit (Kapa Biosystems, Inc), except with 10 μL volume and 90 s annealing/extension PCR. Libraries were pooled and normalized to 4 nM. Pooled libraries were re-quantified by ddPCR on a QX200 system (Bio-Rad), using the Illumina TruSeq ddPCR Library Quantification Kit and following manufacturer’s protocols. The libraries were sequenced in two 2 × 76 bp paired end v3 runs on a MiSeq instrument (Illumina) at 13.5 pM, following the manufacturer’s protocols. FASTQ files were generated for each sample by the MiSeq Instrument Software and used for transcriptomic analysis.

### Transcriptomic Analysis

Reads were mapped to the *C. jejuni* 81-176 genome (Genome: NC_008787, pTet: NC_008790, and pVir: NC_008770) using Bowtie2 (version 2.3.2) and counted with featureCounts (Subread package version 1.5.3). Differential expression was analyzed with DESeq2 (version 1.18.1) in R (version 3.4.2). Two biological replicates were analyzed for each experimental condition and compared against the transcriptome in MH broth from 18 h as the baseline.

### Proteomic Sample Preparation and LC-MS/MS Analysis

*Campylobacter jejuni* strain 81–176 was grown in MH broth for 20 h and the culture was either inoculated in MH broth at OD_540_ of 0.3 (negative sample) or suspended to an OD_540_ of 0.3 in MEM with 1% FBS alone, with INT 407 cells, and with Caco-2 cells for 4 h. For each of the tested conditions, three biological replicates collected on different days were compared for proteomic analysis. Ten milliliters of each sample was harvested by centrifugation at 4°C at 13,000 × *g*, rinsed twice with PBS, then frozen in liquid nitrogen and stored at -80°C prior to further sample preparation. For each sample, the bacterial pellet was suspended in 100 mM ammonium bicarbonate and lysed by bead beating using 0.1 mm zirconia/silica beads for 5 vortexing periods of 1 min separated by rest periods of 30 s on ice. Eight molar urea and 5 mM DTT were added to the samples prior to incubation for 1 h at 37°C. Samples were then diluted eight times prior to trypsin digestion for 3 h at 37°C. Digested peptides were desalted using C18 SPE cartridges (Discovery C18, 1 mL, 50 mg, Supelco). The peptide concentrations were measured by BCA assay (Thermo Scientific).

Five microliters of 0.1 μg/μL were analyzed by reverse phase LC-MS/MS using a Waters nanoEquity^TM^ UPLC system (Millford, MA, United States) coupled with a QExactive HF mass spectrometer from Thermo Fisher Scientific (San Jose, CA, United States). The LC was configured to load the sample first on a solid phase extraction (SPE) column followed by separation on an analytical column. Analytical columns were made in-house by slurry packing 3-μm Jupiter C18 stationary phase (Phenomenex, Torrance, CA, United States) into 70-cm long, 360 μm OD × 75 μm ID fused silica capillary tubing (Polymicro Technologies Inc., Phoenix, AZ, United States). Samples were separated using a 200 min gradient. The effluents from the LC column were ionized by electrospray ionization and mass analyzed with a QExactive hybrid quadrupole/Orbitrap mass spectrometer operated in the data-dependent analysis mode. Top 12 ions from the survey scan were selected by a quadrupole mass filter for high energy collision dissociation (HCD) and mass analyzed by the Orbitrap. An isolation window of 2 daltons was used for the isolation of ions and collision energy of 28% was used for HCD with an AGC setting of 10^5^ ions. Mass spectra were recorded for 200 min by repeating this process with a dynamic exclusion of previously selected ions for 45 s.

### Proteomic Data Analysis

Raw mass spectrometry data were processed using the MaxQuant computational proteomics platform (Cox et al., 2014). The false discovery rate was set at 0.01 at the peptide and protein level. Proteins were identified with at least 2 peptides of a minimum length of 6 amino acids by searching against the RefSeq *Campylobacter jejuni* 81-176 database (Genome: NC_008787, pTet: NC_008790, and pVir: NC_008770; 1,680 sequences). MaxLFQ was used for quantification. The proteins only identified by site, reverse peptides, and potential contaminants were removed for further analysis. Only the proteins with a measured LFQ intensity in at least two out of the three samples of a given condition (i.e., >66% completeness) were used for quantification. The LFQ intensities were then log2 transformed and median normalized within each sample. Statistics (e.g., PCA, *T*-test, ANOVA and Tukey’s HSD tests) were performed in R using the packages Stat, FactoMineR (Le et al., 2008), and Agricolae (Mendiburu and Simon, 2015).

### COG Category Assignment

Genomic and associated plasmid sequences were obtained from RefSeq for *C. jejuni* strain 81-176 (Genome: NC_008787, pTet: NC_008790, and pVir: NC_008770). PSI-BLAST (version 2.2.31+) was used to identify proteins in the *C. jejuni* 81-176 genome that had similarity to proteins in the COG database (Galperin et al., 2015) using an *e*-value cutoff of 1 × 10^-3^. The top PSI-BLAST hit for each gene in *C. jejuni* 81–176 was used to assign the COG category. Genes with no match were given the assignment “Uncategorized.” Testing for COG category enrichment was done using Fisher’s exact test in R (version 3.4.2).

### Generation of *C. jejuni* Deletion Mutants

To generate the *C. jejuni* 81–176 *flgL, flhF, cetAB, cheB*, and *Cj0448c* mutants, the upstream and downstream regions of *flgL, flhF, cetAB, cheB*, and *Cj0448c* were PCR-amplified from *C. jejuni* strain 81–176 using the CloneAmp^TM^ HiFi PCR Premix (Clontech Laboratories, Inc.) and the primers listed in Table 1. To insert the chloramphenicol resistance gene between the upstream and downstream regions of the target genes, the chloramphenicol resistance cassette was PCR-amplified from the *E. coli*-*C. jejuni* shuttle vector pRY111 with primer pairs MEK4533 and MEK4534 (Yao et al., 1993). The *C. jejuni* suicide vector pBSK-Kan2 was used to create the recombinant plasmids for deleting *flgL, flhF, cetAB, cheB*, and *Cj0448c*. The final constructs were generated using the in-fusion cloning strategy with the In-Fusion HD Cloning Kit (Takara Bio USA Inc. Mountain View, CA, United States), as described previously (Gourley et al., 2017). All constructs were confirmed by both restriction digestion and Sanger sequencing. The constructs were electroporated into the *C. jejuni* strain 81-176 to generate the desired mutants through homologous recombination. The transformants were selected on MHB agar supplemented with 8 μg/mL chloramphenicol and checked for gene deletion by PCR.

**Table 1:** Bacterial strains, plasmids, and oligonucleotides used in this study.

Bacterial strains		Description	References
*C. jejuni* F38011			
*C. jejuni* 81–176			Korlath et al., 1985
81–176 Δ*flaAB*		*C. jejuni* 81–176 *flaAB* deletion mutant	Neal-McKinney and Konkel, 2012
81–176 Δ*flhF*		*C. jejuni* 81–176 *flhF* deletion mutant	This study
81–176 Δ*flgL*		*C. jejuni* 81–176 *flgL* deletion mutant	This study
81–176 Δ*cetAB*		*C. jejuni* 81–176 *cetAB* deletion mutant	This study
81–176 Δ*cheBR*		*C. jejuni* 81–176 *cheB* deletion mutant	This study
81–176 Δ*Cj0448c*		*C. jejuni* 81–176 *Cj0448c* deletion mutant	This study
81–176 Δ*flaAB* : *flaAB*		*C. jejuni* 81–176 *flaAB* deletion mutant complemented with wild-type *flaAB* gene	This study
81–176 Δ*flhF* : *flhF*		*C. jejuni* 81–176 *flaAB* deletion mutant complemented with wild-type *flhF* gene	This study
81–176 Δ*flgL* : *flgL*		*C. jejuni* 81–176 *flgL* deletion mutant complemented with wild-type *flgL* gene	This study
*E. coli* HST08		Stellar chemically competent cells	Takara Bio.

**Plasmids**			

**Plasmid ID**	**Description**	**Antibiotic resistance**	**References**

	pBSK-Kan2	Kan^R^	Flanagan et al., 2009
	pRY111	Cm^R^	Yao et al., 1993
	prRNA-Hygro	Kan^R^ Hyg^R^	Gourley et al., 2017
	pBSK-Kan2-*flaAB*-KO	Kan^R^ Cm^R^	Neal-McKinney and Konkel, 2012
	pBSK-Kan2-*flgL*-KO	Kan^R^ Cm^R^	This study
pPKT279	pBSK-Kan2-*flhF*-KO	Kan^R^ Cm^R^	This study
pPKT280	pBSK-Kan2-*cetAB*-KO	Kan^R^ Cm^R^	This study
pPKT281	pBSK-Kan2-*cj0448c*-KO	Kan^R^ Cm^R^	This study
pPKT282	pBSK-Kan2-*cheB*-KO	Kan^R^ Cm^R^	This study
pPKT283	prRNA-Hygro-*flhF*-comp	Kan^R^ Hyg^R^	This study
pPKT287	prRNA-Hygro-*flgL*-comp	Kan^R^ Hyg^R^	This study
pPKT292	prRNA-Hygro-*flaAB*-comp	Kan^R^ Hyg^R^	This study

**Primers**			

**Primer ID**	**Oligo name**	**Sequences 5′-3′*^a^***	**References**

Mutant generation primers			
MEK1866	FlaAB-up-SacI-FW	ATATAGAGCTCAAGAAAGAGTAAATTTACAACTTAGG	Neal-McKinney and Konkel, 2012
MEK1867	FlaAB-up-SacII-RV	ATATACCGCGGAAATAATTTCAAACTCATCCATGAGC	Neal-McKinney and Konkel, 2012
MEK1868	FlaAB-dn-SacII-FW	ATATACCGCGGAAACTATTACAATAATCTTTCTAAAGAGC	Neal-McKinney and Konkel, 2012
MEK1869	FlaAB-dn-XhoI-RV	ATATACTCGAGAATAATAATATAGCAGAGTTAATTTTTGG	Neal-McKinney and Konkel, 2012
MEK3965	FlgL-up-FW	ACACCTGCAGTTTTTTCCTCTAAAGTATTAAAGTTAAAAT	This study
MEK3966	FlgL-up-RV	GGGAACAAAAGCTGGAGCTCGCTAGAAGCTTGGTAAATTCTG	This study
MEK3961	FlgL-dn-FW	TATAGGGCGAATTGGGTACCCTGCTCTATTTCACGCAATA	This study
MEK3962	FlgL-dn-RV	GATCGGATCCAATTTTTTATGGTATAATTTGGCTTTGA	This study
MEK3963	CAT-FlgL-FW	ATAAAAAATTGGATCCGATCTGCGCCCTTTAGT	This study
MEK3964	CAT-FlgL-RV	GAGGAAAAAACTGCAGGTGTTCCTTTCCAAGTTAATTG	This study
MEK4355	FlhF-up-XhoI-FW	GGGCCCCCCCTCGAGCAAATGAAACTTGCAGCAT	This study
MEK4356	FlhF-up-SacII-RV	GGAACACCCGCGGATATATTCCTTATATCATGCCTAAAGGCA	This study
MEK4357	FlhF-dn-SacII-FW	AAAGGGCCGCGGATTATTTAGAAGTAGCCAATAG	This study
MEK4358	FlhF-dn-SacI-RV	CAAAAGCTGGAGCTCAGCGCTTAAATGACCTAAGAA	This study
MEK4359	Cj0448c-up-XhoI-FW	GGGCCCCCCCTCGAGAAGGTCCAAAAGTCAAATTTG	This study
MEK4360	Cj0448c-up-SacII-RV	GGAACACCCGCGGATAGCCTTATTTTTTATAATTTGC	This study
MEK4361	Cj0448c-dn-SacII-FW	AAAGGGCCGCGGATTAGGTTGAACCTTTTTAAG	This study
MEK4362	Cj0448c-dn-SacI-RV	CAAAAGCTGGAGCTCATTATACAGAAGGAAGAAGTT	This study
MEK4363	CheB-up-XhoI-FW	GGGCCCCCCCTCGAGGATGTAAGGTCTGCTTTGAT	This study
MEK4364	CheB-up-SacII-RV	GGAACACCCGCGGATATGAAATTTTATTTTTCTTGTCACGTA	This study
MEK4365	CheB-dn-SacII-FW	AAAGGGCCGCGGATTTTAAAACCTATGAGCTTA	This study
MEK4366	CheB-dn-SacI-RV	CAAAAGCTGGAGCTCCAACATAAAGCCTTCCTT	This study
MEK4367	CetAB-up-XhoI-FW	GGGCCCCCCCTCGAGCACTCCTTTTGATCTTTG	This study
MEK4368	CetAB-up-SacII-RV	GGAACACCCGCGGATAGAAGTCATAAATCAAAACAATAC	This study
MEK4369	CetAB-dn-SacII-FW	AAAGGGCCGCGGATTACTTATAATGAGCTAATCTT	This study
MEK4532	CetAB-dn-SacI-RV	CAAAAGCTGGAGCTCCACCTTCAATCTGTCCTGTA	This study
MEK4533	CAT-SacII-FW	TATCCGCGGGTGTTCCTTTCCAAGTTAATTGC	This study
MEK4534	CAT-SacII-RV	AATCCGCGGCCCTTTAGTTCCTAAAGG	This study
Complementation primers			
MEK4545	FlhF-prom-Comp-XbaI-FW	GATCACCTCCTTTCTAGACGAATTTTTAGGTGTTTTGATGATTTTAGC	This study
MEK4546	FlhF-Prom-Comp-RV	GTTGTCCCATGGATTTAACCTTAAAAATTTATTTTTAACCTTTTATTATAAC	This study
MEK4547	FlhF-ORF-Comp-FW	GGTTAAATCCATGGGACAACTTATACATACTTTTACTGTTGAAGATAC	This study
MEK4548	FlhF-ORF-Comp- 3 × FLAG-BamHI-RV	GAGCTTTGAATTCGGATCCTTATTTATCATCATCATCTTTATAATCAATATCATGATCTTTATAATCA CCATCATGATCTTTATAATCTTCATTATTTTTTCCTTTGTTAAACCCTTCTAAAATACAATG	This study
MEK4557	FlgL-Comp-XbaI-FW	CACCTCCTTTCTAGAAGGCTAAAAAATACTTTAAAAATAAAAAGTATTTTCAAAAAGACG	This study
MEK4558	FlgL-Comp-BamHI-3 × FLAG-RV	CAAGAGCTTTGAATTCGGATCCTTATTTATCATCATCATCTTTATAATCAATATCATGATCTTTATAA TCACCATCATGATCTTTATAATCCATATAATTTAATAAGCTAAGTTGAGAAATTGTTGTACTAGC	This study
MEK4561	FlaAB-Comp-XbaI-FW	GGATCACCTCCTTTCTAGAGAGTGAAGTTATTGTTAGTAAAATTGAAGATG	This study
MEK4562	FlaAB-Comp-BamHI-RV	CAAGAGCTTTGAATTCGGATCCCCTTAATTGAAACTATAATAGATCTTATAGAAAGTC	This study


*^*a*^Underline marks indicate the restriction sites in the primer sequence.*

### Generation of *C. jejuni* Complemented Isolates

The *C. jejuni* 81–176 *flhF, flaAB*, and *flgL* mutants were complemented using homologous recombination to insert genes into the rRNA gene cluster. Because *aroQ* is predicted to be the first gene in the operon that contains *flhF* ^1^, the promoter sequence of *aroQ* was amplified and fused with the *flhF* ORF with a 3 × FLAG tag. To complement the *flaAB* mutant, a 3988-bp DNA fragment containing the promoter and ORF of *flaAB* was PCR-amplified. To complement the *flgL* mutant, a 2459-bp DNA fragment containing the promoter and ORF of *flgL* with a 3 × FLAG tag was PCR-amplified. The PCR products were cloned into the *C. jejuni* suicide vector prRNA-Hygromycin using the In-Fusion cloning strategy, as described previously (Gourley et al., 2017). All constructs were confirmed by restriction-digestion and Sanger-sequencing. All transformants were selected on MHB agar supplemented with 250 μg/mL hygromycin and confirmed by PCR. Antibodies specific for the FLAG tag and FlaAB were used to confirm protein synthesis in the complemented isolates.

### Motility Assay and Flagellar Stain

Motility was assessed by spotting 3 μL of the relevant *C. jejuni* culture suspended at an OD_540_ of 0.1 on a MH soft-agar plate (0.4% agar). Images were captured with a GE ImageQuant LAS-4000 mini and measured in ImageJ. Flagella were stained using Flagella Stain (Hardy Diagnostics) following the directions from the manufacturer. Images of the stained *C. jejuni* were acquired with a Nikon Eclipse TE2000-U microscope equipped with a Photometrics Coolsnap HQ camera. Flagella were measured and quantified in ImageJ. Only bacteria where both ends were clearly visible were measured.

### Statistical Analysis

On charts, *p*-values were calculated using GraphPad Prism 6.0 g (GraphPad Software, La Jolla, CA, United States) using the statistical test indicated in the figure legends.

### Accession Numbers

RNA-Seq data have been deposited in the NCBI Gene Expression Omnibus database with the identifier GSE114909. The mass spectrometry proteomics data have been deposited in the ProteomeXchange Consortium via the PRIDE (Vizcaino et al., 2016) partner repository with the dataset identifiers PXD009817 and 10.6019/PXD009817. Processed data (fold-changes and *p*-values) for the proteomics experiment is available in Supplementary Table S1, and data for the RNA-Seq experiment is available in Supplementary Table S2.

## Results

### Protein Synthesis Is Required for Maximal Invasion of Host Cells by *C. jejuni*

#### *C. jejuni* Must Be Metabolically Active to Invade Host Cells

**FIGURE 1:**
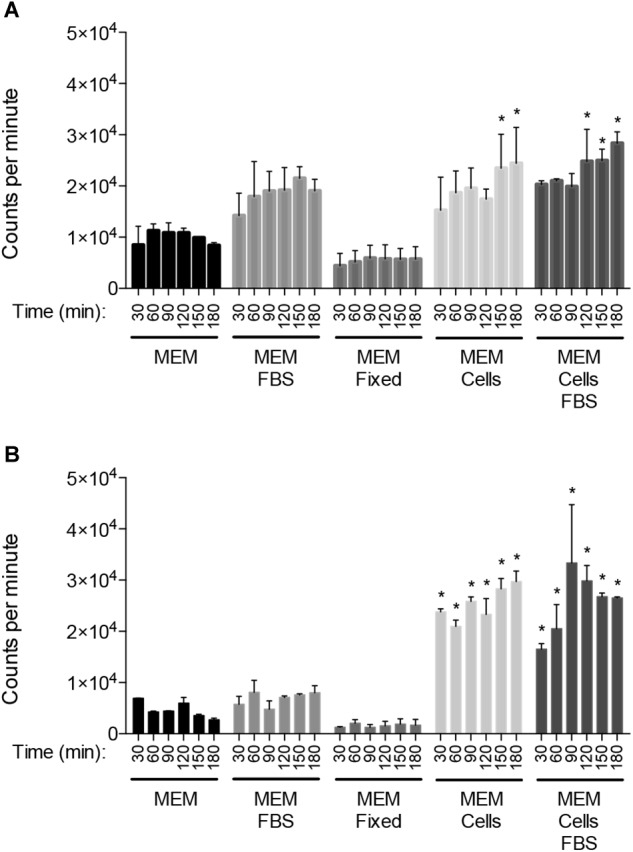
Co-cultivation of *C. jejuni* with viable epithelial cells stimulates enhanced metabolic activity. Temporal kinetics of [^35^S]-methionine incorporation by *C. jejuni* strains 81–176 **(A)** and F38011 **(B)** incubated in MEM, MEM plus 1% FBS, MEM with human INT 407 epithelial cells that had been fixed with paraformaldehyde, MEM with viable human INT 407 epithelial cells, and MEM plus 1% FBS and viable human INT 407 epithelial cells. The FBS used in these experiments was albumin-depleted and dialyzed against PBS. The [^35^S]-methionine labeling was performed in the presence of emetine hydrochloride to prevent radioactive methionine incorporation by the host cells. The bacteria were pelleted at the end of the time intervals indicated, washed in PBS, and amount of [^35^S]-methionine incorporation determined by measuring counts per minute following trichloroacetic acid (TCA) precipitation. Shown is the mean ± the standard deviations. Significant differences from the MEM alone at each time point were determined by one-way ANOVA followed by Sidak’s multiple comparisons test (^∗^*p* < 0.05).

To account for possible phenotypic variation, this study compared two similar, but not identical (94% genomic identity), *C. jejuni* strains with demonstrated human virulence potential. *C. jejuni* strain 81-176 and strain F38011 were recovered from individuals with diarrhea containing blood and leukocytes in the stool. We assessed the metabolic activity of *C. jejuni* strains 81-176 and F38011 to determine if each strain elicits enhanced metabolic activity when cultured with epithelial cells (Figure 1). In contrast to *C. jejuni* incubated in MEM or MEM supplemented with FBS, the metabolic activity of the two *C. jejuni* strains was significantly increased when each was co-cultivated with viable INT 407 epithelial cells (*p* < 0.05). Fixation of the epithelial cells with paraformaldehyde before performing the metabolic labeling assay failed to stimulate enhanced *C. jejuni* metabolic activity, indicating that the host cell-stimulatory molecule is sensitive to fixation. This result indicates that viable host cells are necessary to stimulate *C. jejuni* metabolic activity.

#### Temporal Kinetics of *C. jejuni* Internalization

**FIGURE 2:**
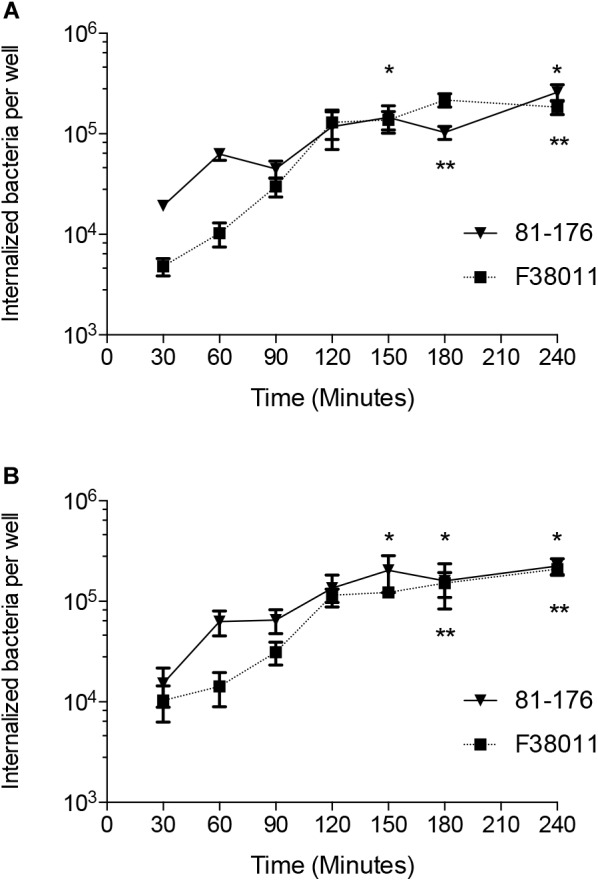
The number of internalized *C. jejuni* increases significantly over time. The temporal kinetics of *C. jejuni* internalization was determined using INT 407 cells **(A)** and Caco-2 cells **(B)**. Values represent number of gentamicin-protected bacteria/well of 24-well tissue culture tray and are given as means of triplicate determinations ± the standard deviations of *C. jejuni* strain 81–176 (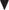) and *C. jejuni* strain F38011 (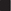). Significant differences from the 30 min time point were determined by a Kruskal–Wallis test followed by Dunn’s multiple comparisons test for each strain individually (^∗^81–176, ^∗∗^F38011, *p* < 0.05).

INT 407 cell monolayers were inoculated with *C. jejuni* strains 81-176 and F38011 to examine the kinetics of internalization. Consistent with previous work, no appreciable differences were detected in the total number of cell-associated (adherent) *C. jejuni* over the course of the 3 h assay (not shown) (Konkel et al., 1993). However, the number of internalized (gentamicin-protected) *C. jejuni* increased by about two orders of magnitude during the interval from 30 to 180 min (Figure 2). Moreover, a 12-fold increase in the number of *C. jejuni* internalized was observed from the 1 and the 2.5 h time points with both INT 407 and Caco-2 cells (*p* < 0.05, Supplementary Figure S1). The observed increase is not due to bacterial replication, as the doubling time of both *C. jejuni* strains is more than 2 h under ideal conditions (MH broth). These findings indicate that the kinetics of two *C. jejuni* clinical strains are similar to one another and that invasion potential of both strains is similar for human INT 407 and Caco-2 epithelial cells.

**FIGURE 3:**
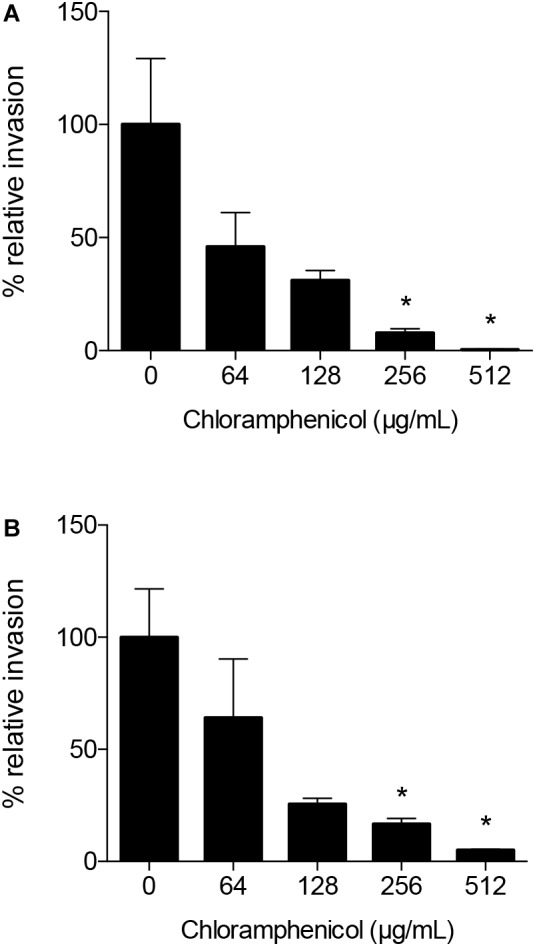
Chloramphenicol, a selective inhibitor of bacterial protein synthesis, significantly reduces the number of *C. jejuni* internalized by human INT 407 cells in a dose-dependent manner. The epithelial cells were inoculated with *C. jejuni* strain 81–176 **(A)** and *C. jejuni* strain F38011 **(B)** suspended in media with and without (untreated control) chloramphenicol to determine if *C. jejuni*-cell invasion is dependent on *de novo* protein synthesis. Following the 3 h incubation period to allow *C. jejuni* to adhere to and invade the epithelial cells, the cells were rinsed and gentamicin was added to kill the extracellular bacteria. Control experiments revealed that the concentrations of chloramphenicol used in these assays had no detectable quantitative or qualitative effect on INT 407 cell protein synthesis, as judged by determination of total [^35^S]-methionine incorporation into protein and by sodium dodecyl sulfate-polyacrylamide gel electrophoresis and fluorography, respectively. In addition, chloramphenicol had no detectable effect on bacterial viability. The number of gentamicin-protected bacteria/well of a 24-well tissue culture tray was determined by direct plate counts after lysing the monolayers with a solution of 0.1% Triton X-100. Values represent the percent relative invasion, where 100% is set to the invasion for the untreated control (number of gentamicin-protected bacteria/well of a 24-well tissue culture tray for the untreated control) ± the standard deviations. Significant differences from the non-treated control were determined by a Kruskal–Wallis test followed by Dunn’s multiple comparisons test (^∗^*p* < 0.05).

To determine if the increase observed in *C. jejuni*-cell invasion is dependent on *de novo* protein synthesis, the gentamicin-protection assay was performed in the presence of chloramphenicol. Chloramphenicol is a selective inhibitor of bacterial protein synthesis (Konkel and Cieplak, 1992). Chloramphenicol significantly reduced the number of *C. jejuni* internalized by the INT 407 cells in a dose-dependent manner (Figure 3). The chloramphenicol treatment had no detectable effect on the viability of the *C. jejuni* strains over the course of the invasion assay (not shown). Collectively, these data demonstrate *C. jejuni* is metabolically active and must be able to synthesize proteins to efficiently invade human epithelial cells.

### Proteomic and Transcriptomic Analyses of *C. jejuni* Cultivated With Epithelial Cells Unmask Putative Virulence Genes/Proteins

The results of the invasion assays indicated that upon co-culture with epithelial cells, *C. jejuni* synthesizes proteins that promote host cell invasion. To identify the genes and proteins that either enhance or promote this phenotype, we analyzed the proteomic and transcriptomic profiles of *C. jejuni* co-cultured with human epithelial cells. *C. jejuni* strain 81-176 was selected for these studies because it is commonly used in other laboratories. Please note that the gene designation (locus tag) numbers listed in all tables are provided for both *C. jejuni* strains 81–176 and the NCTC 11168 homolog, but we have opted to list the gene designation using strain NCTC 11168 throughout the text given the frequency of usage in the literature.

#### Proteomic Analysis of *C. jejuni* Cultivated With Epithelial Cells

**FIGURE 4:**
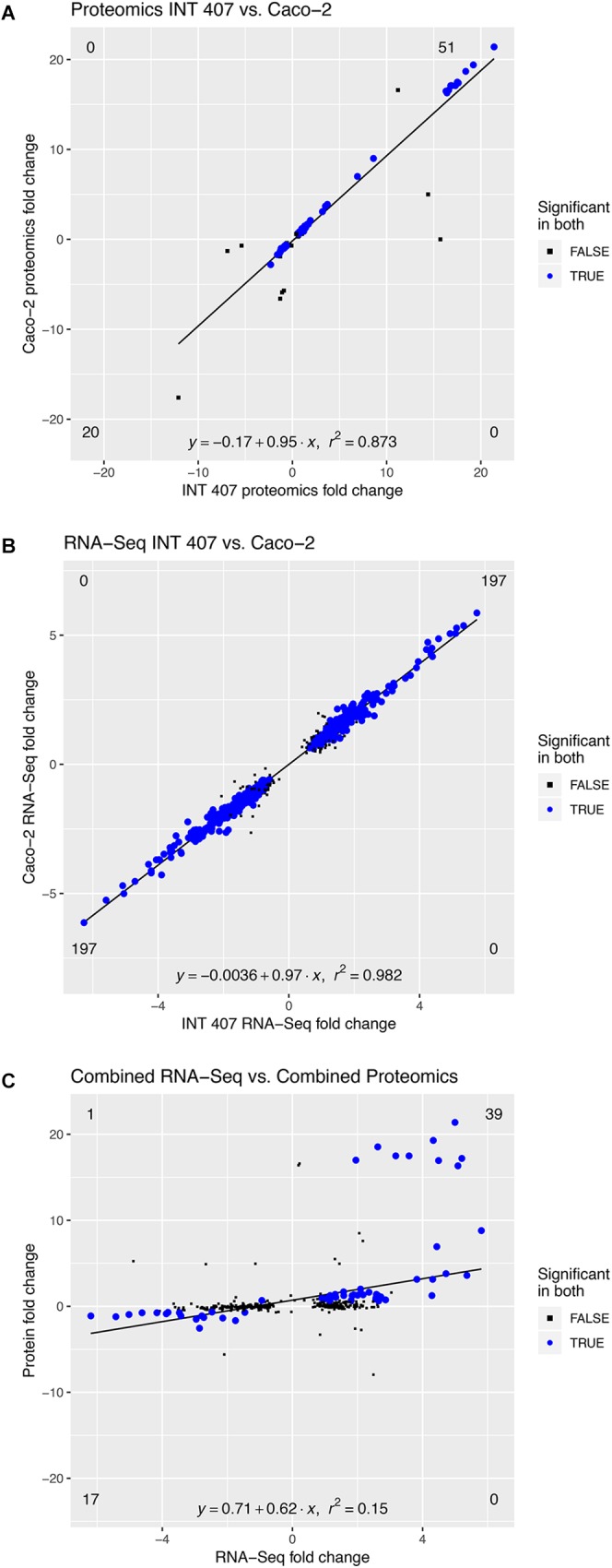
The *C. jejuni* response to INT 407 and Caco-2 cells at 4 h is similar on the proteomic and transcriptomic level. Panels: **(A)** Protein profiles of *C. jejuni* co-cultured with human INT 407 and Caco-2 epithelial cells were compared. **(B)** RNA-Seq profiles of *C. jejuni* co-cultured with human INT 407 and Caco-2 epithelial cells were compared. **(C)** The proteins and genes from **(A,B)** that were significantly altered in both conditions (INT 407 and Caco-2, blue dots) were compared. Due to the high correlation between the INT 407 and Caco-2 transcript and protein profiles, the fold-change of the two host cell types was averaged.

Proteomic analysis was performed using three biological replicates of *C. jejuni* strain 81-176 co-cultured with epithelial cells for 4 h, and the data were compared to *C. jejuni* growth in MH broth for 4 h. We chose to use the INT 407 and Caco-2 cells because these two cell lines have been used to study *C. jejuni*-host cell interactions for more than two decades (McSweegan and Walker, 1986; Everest et al., 1992; Konkel and Cieplak, 1992; Friis et al., 2005). Comparisons were made between the responses to INT 407 cells and Caco-2 cells (see Figure 4A). Proteins that were significantly altered [log_2_(fold-change) > 0.6, *p* < 0.05] were compared between the two co-incubation conditions (INT 407 and Caco-2) and there was a high Pearson’s correlation (*r*^2^ = 0.873). Based on analysis of the proteomic data, *C. jejuni* responded to co-culture with INT 407 and Caco-2 cells in a similar fashion (Supplementary Figure S2 and Supplementary Table S1). More specifically, only 15 of the 105 differentially abundant proteins (14.2%) had fold-changes that were more than 20% different between the INT 407 and Caco-2 cells. Noteworthy is that 14 of the 15 *C. jejuni* proteins whose abundance changed (increased or decreased) with the INT 407 cells, also increased or decreased in abundance with the Caco-2 cells. The one exception was Cj0062c, which was detected in *C. jejuni* co-cultured with Caco-2 cells but not detected in *C. jejuni* co-cultured with INT 407 cells. Cj0062c is annotated as a putative integral membrane protein and may be required for motility (Hendrixson et al., 2001). The total number of proteins found to be significantly altered in each cell type were similar: 63 increased and 25 decreased in co-culture with INT 407 cells and 58 increased and 30 decreased in co-culture with Caco-2 cells (Supplementary Figure S2). Also apparent was that the proteins with the greatest fold change (>10-fold) were those increasing in abundance, rather than decreasing in abundance. Among these proteins that had the greatest change were the hemin uptake proteins ChuABC (17–21 fold increase).

#### Transcriptomic Analyses of *C. jejuni* Cultivated With Epithelial Cells Revealed Putative Virulence Genes

To gain further insight into the responses of *C. jejuni* to co-culture with epithelial cells, we performed RNA-Seq on *C. jejuni* strain 81–176 co-cultured with INT 407 and Caco-2 cells. RNA was extracted from the *C. jejuni* used to inoculate the cell cultures (time = 0) and from *C. jejuni* co-cultured with epithelial cells for 2.5 and 4 h, which corresponds to the stage at which the bacteria are rapidly invading the cells (see Supplementary Figure S1). It is important to note that the number of the *C. jejuni* co-cultured with epithelial cells does not appreciably increase from 2.5 to 4 h. Genes that were differentially expressed during co-incubation with both cell types were compared at the 2.5 and 4 h time points and found to be similar to one another (Supplementary Figures S3, S4 and Supplementary Table S2). More specifically, comparison of the differentially expressed genes during co-culture with the INT 407 and Caco-2 cells revealed that only 37 of the 512 genes (7.2%) demonstrated fold-changes that were more than 20% different between them. Even though fold-changes were observed in the expression of these genes between INT 407 and Caco-2 cells, their expression levels changed in the same direction (i.e., the genes upregulated in response to the INT 407 cells were also upregulated in response to the Caco-2 cells, and *vice versa*). Throughout the remainder of this manuscript, we will use the terms upregulated gene expression and downregulated gene expression to refer to greater or fewer RNA sequencing reads detected for a specific transcript, without implying any mechanistic basis for gene regulation. In total, there were a total of 197 genes that were upregulated and 197 genes that were downregulated in both INT 407 and Caco-2 cells at the 4 h time point. In summary, the gene expression profiles of *C. jejuni* co-cultured with the INT 407 and Caco-2 cells were similar (*r*^2^ = 0.982) (Figure 4B).

A common theme shared among pathogenic bacteria is to modulate gene expression profiles in the host, resulting in an increased amount of virulence proteins during the course of an infection. Therefore, we examined the genes whose expression increases when *C. jejuni* are co-cultured with epithelial cells (Supplementary Table S2). Inspection of the list of upregulated genes clearly indicates that a subset of these genes encode products that coordinate pathogenesis-related phenotypes. We observed an upregulation of oxidative stress response genes (i.e., *ahpC, katA*, and *sodB*) that have previously been demonstrated to be important for chicken colonization (Palyada et al., 2009), adhesion and invasion-related genes (i.e., *ciaB* and *peb3*) that facilitate cellular invasion, and iron acquisition genes (*cfbpABC, ceuBCDE*, and *chuABCD*) that are critical for bacterial growth and host colonization. Furthermore, we observed an overlap with previous studies investigating the bile salt deoxycholate, particularly in the genes related to oxidative stress response (Negretti et al., 2017). Collectively, the analysis revealed that *C. jejuni* upregulates genes encoding products that contribute to survival in the host cell environment and that facilitate host cell interactions.

#### Comparison of Proteomic and Transcriptomic Data

The proteomic and transcriptomic data generated from the *C. jejuni* co-cultured with INT 407 and Caco-2 cells were compared at the 4 h time point to identify trends (Figure 4C). Similar increases and decreases were observed in protein levels and gene transcripts between the proteomic and transcriptomic data sets (Supplementary Table S3). Moreover, a relationship of increased protein and transcript levels was also apparent, where the proteins that were increased more than 10-fold in abundance had corresponding transcripts that were present in greater levels (Figure 4C and Supplementary Table S3). For example, the uncharacterized genes *Cj1383c* and *Cj1384c* were among the ten with the highest transcript level (>4.5-fold) and protein level (>16-fold) changes. Thus, the correlation between proteomic and transcriptomic data sets provided confidence that the appropriate methodologies were being applied to dissect the bacterium’s adaptation to co-culture with host cells.

Based on the premise that the genes encoding products that promote bacteria-host cell interactions increase over time, analyses were performed to identify the genes upregulated from 2.5 to 4 h in both INT 407 and Caco-2 cells. This list of differentially expressed genes were then compared with the list of proteins detected in greater abundance when *C. jejuni* was co-cultured with the INT 407 and Caco-2 cells for 4 h (Supplementary Table S4). Similar to the data generated from the 4 h time point, many proteins and genes were identified whose protein levels and transcript levels changed in a similar manner. Twenty seven genes were identified whose expression increased from 2.5 to 4 h in both INT 407 and Caco-2 cells and five genes were identified whose expression decreased from 2.5 to 4 h in both INT 407 and Caco-2 cells. This finding revealed *C. jejuni* responds to co-culture with epithelial cells by upregulating more genes than are downregulated.

**FIGURE 5:**
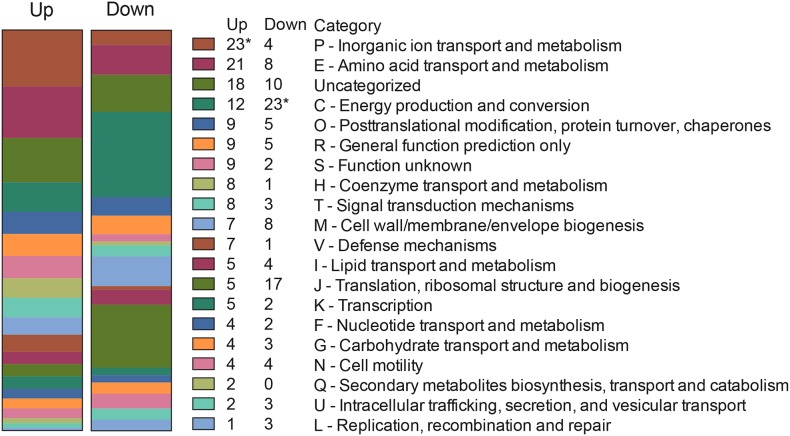
Clusters of Orthologous Groups (COG) categories of *C. jejuni* genes upregulated and downregulated from 2.5 to 4 h in response to co-culture with INT 407 and Caco-2 epithelial cells. Differentially expressed genes (Supplementary Table S5) were categorized by performing a PSI-BLAST against proteins in the COG database, genes with no match in the database were described as “Uncategorized.” Asterisk (^∗^) indicates statistically significant enrichment in that category as determined by Fisher’s exact test (Benjamani–Hochberg adjusted *p* < 0.05).

**FIGURE 6:**
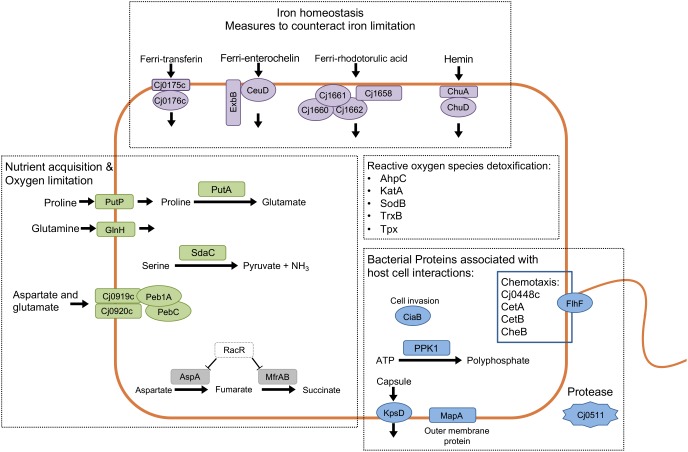
Analysis of the responses of *Campylobacter jejuni* co-culture with epithelial cells using proteomic and transcriptomic approaches provides insight into *C. jejuni*-host cell interactions. Prominent upregulated genes and proteins included iron homeostasis proteins (purple), proteins related to nutrient acquisition and utilization (green), bacterial proteins involved with host-cell interaction (blue), and proteins related to oxygen/nitrogen species detoxification. Indicated in gray are two downregulated genes that are part of the RacRS regulon. Together, these findings indicate that *C. jejuni* responds to epithelial cells by producing products that contribute to its survival and that further promote host cell interactions.

We then analyzed the Clusters of Orthologous Groups (COG) categories of the differentially expressed genes in the RNA-Seq dataset (see Figure 4B) to gain further insight into *C. jejuni*-host cell interactions. We propose this analysis is warranted based on the following observations: (1) similar trends were noted in the proteome and transcriptome analysis of *C. jejuni* cultured with the INT 407 and Caco-2 cells; and (2) genes were identified whose expression increased from 2.5 to 4 h in both INT 407 and Caco-2 cells. In total, 141 genes were identified that belonged to a variety of COG categories (Supplementary Table S5 and Figure 5). Many of the genes identified that were differentially expressed by *C. jejuni* in response to co-culture with epithelial cells are involved in inorganic ion transport, amino acid transport, and metabolism (Figure 5). There were number of genes identified that either belonged to the ‘uncategorized’ or ‘function unknown’ categories (Figure 5), which also may play a role in host cell interaction. Based on the data, it is evident that *C. jejuni* adapts to co-culture with epithelial cells by synthesizing proteins to acquire nutrients, scavenge for inorganic molecules, resist reactive oxygen/nitrogen species, and promote host cell interactions. Figure 6 shows a model for the actions of proteins involved in promoting *C. jejuni* survival and host cell interaction.

### Assessment of Identified Proteins in Host Cell Interaction

#### Motility and Flagella in *Campylobacter* Mutants

**FIGURE 7:**
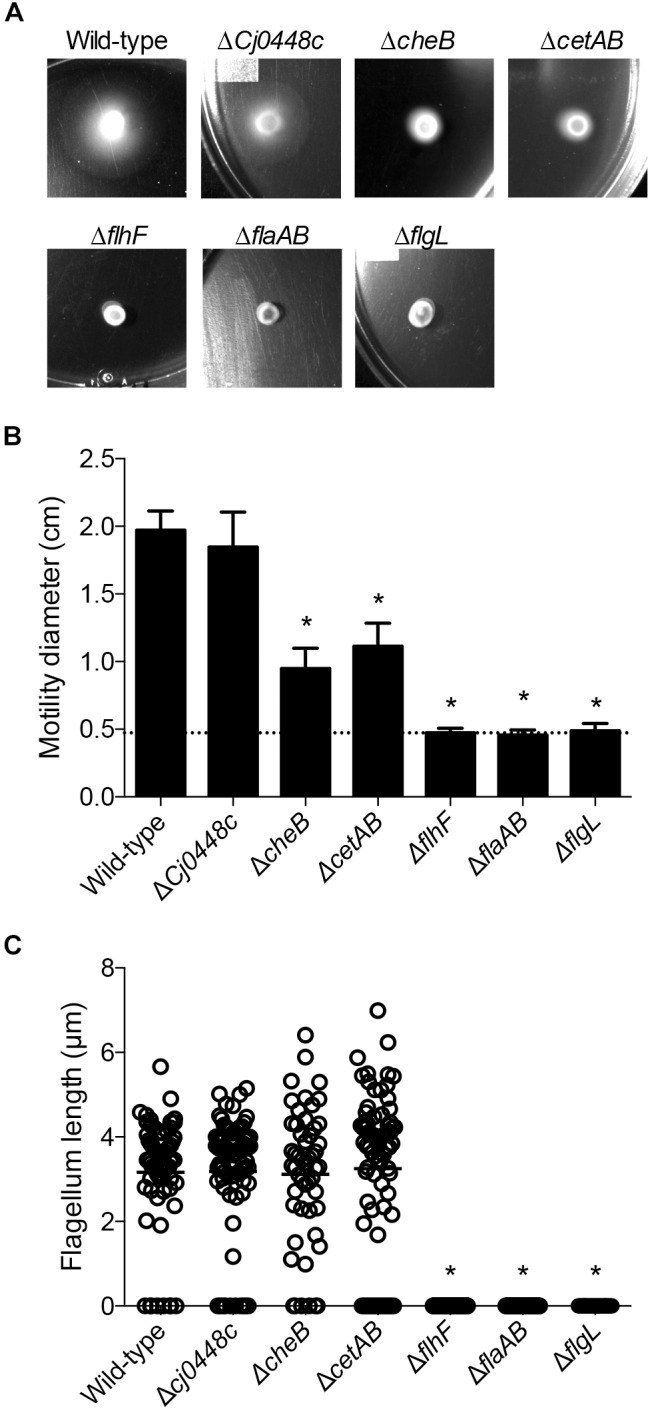
Motility of *C. jejuni* chemotaxis mutants was impaired, and the motility of flagellar biosynthesis mutants was abolished. Motility was tested by spotting a constant amount of *C. jejuni* onto soft agar plates **(A)** and measuring the diameter of the resulting bacterial swarm **(B)**. Shown is the mean ± the standard deviations, with the dotted line indicating the zone of bacterial grown resulting from the spot. The presence and length of the flagellar filament was also determined by staining the flagellum **(C)**. Each measurement is represented by a circle (*n* > 35 cells measured per isolate) with the mean represented by a bar. Significant differences from wild-type were determined by a Kruskal–Wallis test followed by Dunn’s multiple comparisons test (^∗^*p* < 0.05).

Among the genes that were upregulated in the presence of host cells included those involved in chemotaxis and in the regulation of flagellar assembly. Isogenic *C. jejuni* mutants in *Cj0448c, cheBR, cetAB, flhF, flaAB*, and *flgL* were created, and the motility of the isolates was evaluated. Please note that the strategy used for generating the *cheB* gene deletion resulted in a polar mutation on *cheR*, therefore we refer to this isolate as a *cheBR* mutant. In these assays, *C. jejuni flaAB* and *flgL* mutants were included as negative controls, as deletion of the FlaAB or FlgL proteins disrupts motility. The motility of the *Cj0448c* mutant was indistinguishable from the wild-type strain (*p* = 0.77), while the zone of motility of the *cheBR* and *cetAB* mutants was about half that of the wild-type strain (*p* < 0.05). The *flhF, flaAB* and *flgL* mutants were non-motile (*p* < 0.05, Figure 7A,B). Using flagella staining, we observed that the number and length of flagella in the wild-type strain, *Cj0448c, cheBR*, and *cetAB*, mutants were indistinguishable (i.e., >95% had bipolar flagella with an average length of 3.18 μm). In the *flhF* mutant, <5% of the *C. jejuni* had a single flagellum, and no bipolar flagella were observed. No flagella were observed in the *flaAB* and *flgL* mutants (Figure 7C).

#### Cellular Adherence and Invasion of *Campylobacter* Mutants

**FIGURE 8:**
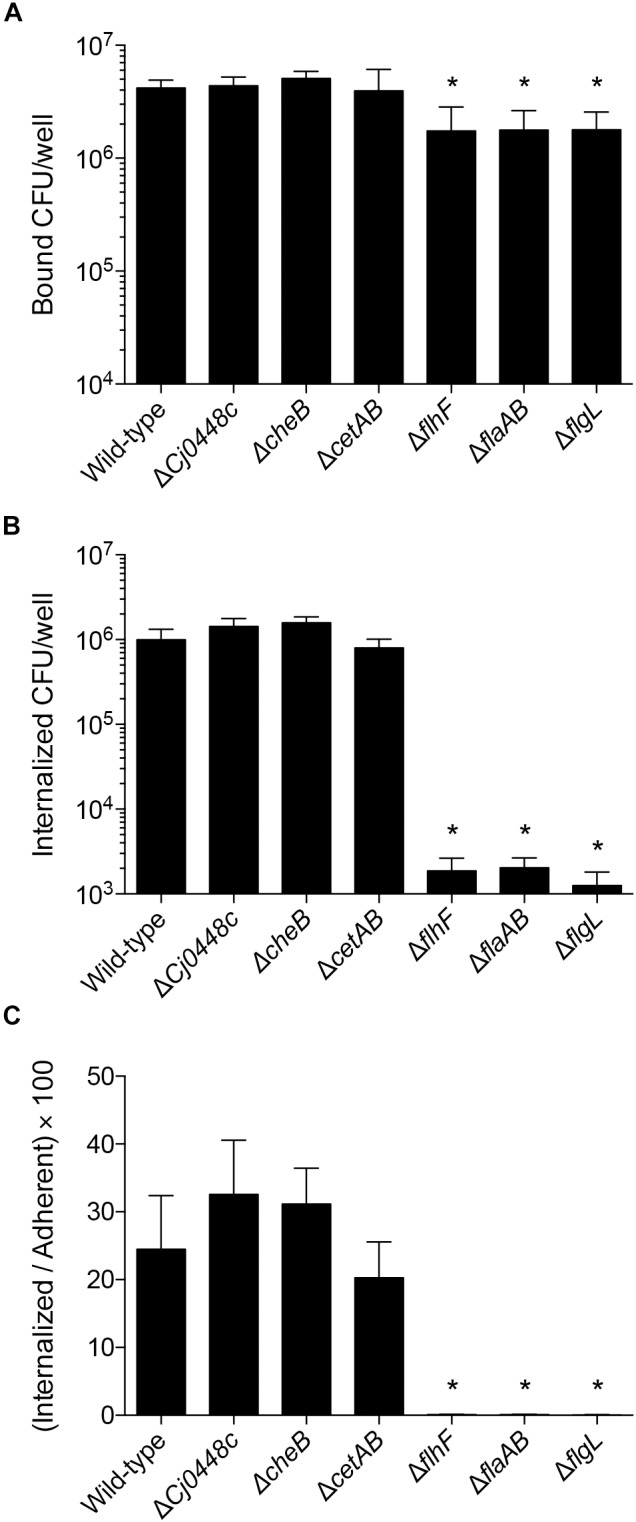
Deletion of chemotaxis genes in *C. jejuni* does not impair invasion of human INT 407 cells, whereas flagellar regulatory and structural mutants are severely attenuated when compared to the wild-type strain. Human INT 407 cells were infected with *C. jejuni* for 3 h, and the number of adherent **(A)** and internalized **(B)** bacteria were determined using the gentamicin-protection assay. Compared to the wild-type strain, the *Cj0448c, cheBR*, and *cetAB* mutants are not deficient in cell adherence or invasion. In fact, the *Cj0448c* and *cheBR* show a small but measurable increase in cellular invasion. In contrast, the *flhF, flaAB*, and *flgL* flagellar mutants are deficient in cellular adherence and severely impaired in cellular invasion. The number of internalized bacteria divided by the number of adherent bacteria was also calculated **(C)**. Shown is the mean ± the standard deviations. Significant differences from wild-type were determined by a Kruskal–Wallis test followed by Dunn’s multiple comparisons test (^∗^*p* < 0.05).

To test the hypothesis that the genes encoding proteins involved in chemotaxis and in the regulation of flagellar assembly promote the interaction of *C. jejuni* with host cells, adherence and internalization assays were performed with the *C. jejuni* mutants and INT 407 cells. The *C. jejuni Cj0448c, cheBR*, and *cetAB* gene deletion mutants did not show a defect in adherence or invasion when compared to a wild-type strain (Figure 8A,B). In fact, there was a small but measurable increase in the invasiveness of the *Cj0448c* and *cheBR* mutants. These findings indicate that these chemotaxis-related proteins are not required for the process of host cell invasion.

Analysis of the *C. jejuni flhF, flaAB*, and *flgL* mutants, in conjunction with the chemotaxis mutants, provided a unique view of the proteins necessary for host cell invasion. While the *flhF, flaAB*, and *flgL* mutants were minimally reduced in cellular adherence, significant reductions were observed in cell invasion compared to the wild-type strain. Similar to the *C. jejuni flaAB* and *flgL* mutants (invasion-negative controls), the *flhF* mutant exhibited more than a 2-log reduction in cell invasion compared to the wild-type strain (Figure 8B, *p <* 0.05). Although small differences in host cell adherence may influence an isolate’s invasive potential, the I/A × 100 value [(number of internalized bacteria divided by the number of adherent bacteria) × 100] demonstrates that the *flhF, flaAB*, and *flgL* mutants are severely impaired in cell invasion (Figure 8C). Collectively, the data demonstrate that the intact flagellum is required for maximal host cell invasion by *C. jejuni*.

#### Complementation of Invasion in *Campylobacter* Mutants

**FIGURE 9:**
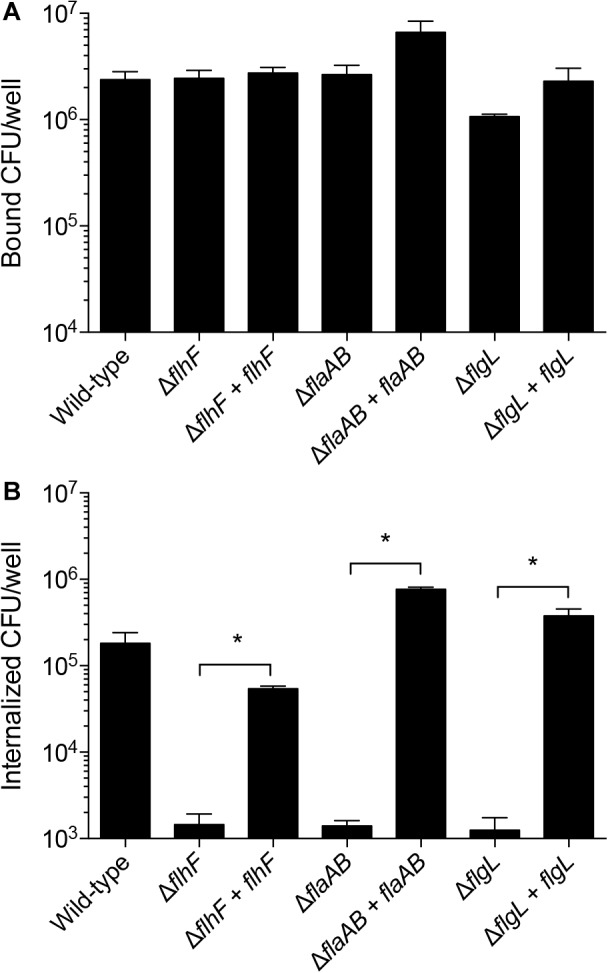
Complementation of the *C. jejuni flhF, flaAB*, and *flgL* genes *in cis* restores cell invasion. Shown is the mean ± the standard deviations. Human INT 407 cells were infected with *C. jejuni* for 3 h, and the number of adherent **(A)** and internalized **(B)** bacteria were determined using the gentamicin-protection assay. Complementation of the *flhF, flaAB*, and *flgL* mutants restored the invasion of these isolates. Significant differences between a mutant and it’s complemented isolate were determined by a Mann–Whitney *U* test (^∗^*p* < 0.05).

*Campylobacter jejuni* mutants with apparent phenotypic differences in cellular invasion compared to the wild-type strain were complemented by inserting the relevant gene(s) in the chromosome within the rRNA gene cluster. Complementation of the *flhF, flaAB*, and *flgL* mutants restored the motility and flagellar length of the isolates to a level indistinguishable from the wild-type strain (not shown). All isolates were similarly adherent to host cells (Figure 9A), and the complementation of the *C. jejuni flhF, flaAB*, and *flgL* isolates restored cellular invasiveness to a level similar to that of the wild-type strain (Figure 9B).

## Discussion

A number of pathogenic bacteria, including *Campylobacter, Salmonella*, and *Shigella*, target the intestinal mucosa and invade gastrointestinal cells. *C. jejuni* is a highly motile pathogen that breaches the mucus layer and then penetrates the physical barrier of the intestinal epithelium by migrating between the cells (Backert and Hofreuter, 2013). In the process, cellular tight junctions are disrupted by the action of bacterial proteases, including a serine protease termed HtrA, which are delivered to the epithelial cells via OMVs (Elmi et al., 2016). *C. jejuni* then adheres to the basolateral surface of the cells via the CadF and FlpA fibronectin-binding proteins, setting the stage for cellular invasion (Konkel et al., 1997, 2010; Larson et al., 2013). The binding of *C. jejuni* to extracellular matrix components allows for the bacteria to manipulate components of cellular focal adhesions, triggering cellular signaling cascades and stimulating cytoskeletal rearrangements. *C. jejuni* invasion of cells results in the production of pro-inflammatory chemokines and cytokines, such as IL-8 (Eucker et al., 2014). While this proposed model of acute disease is supported by the published literature, the ability of *C. jejuni* to respond to epithelial cells is remarkably complex and still incompletely understood. Here we show that *C. jejuni*, a leading bacterial cause of human diarrheal illness worldwide, responds to host cells by altering global protein levels and gene transcription in a manner consistent with a strategy to engage this potentially stressful environment.

Bacteria have developed a remarkable set of systems to sense and appropriately respond to environmental conditions in order to survive (Candon et al., 2007; Sulaeman et al., 2012; Butcher et al., 2015; van der Stel et al., 2015). As such, epithelial cell co-cultures are increasingly being used to study the behavior of infectious microbes in response to host cells during infection. This study was initiated with the intent of identifying the genes expressed and proteins synthesized by *C. jejuni* upon co-culture with epithelial cells. We tried to mimic the host environment by co-culturing *C. jejuni* with human epithelial cells, namely INT 407 and Caco-2 cells. The use of INT 407 and Caco-2 cells was driven, in part, by a desire to understand if different host cells stimulate a conserved or different metabolic, gene expression, or proteosynthetic response from *C. jejuni* during cell culture infections. INT 407 cells were originally derived from the jejunum and ileum of a 2-month-old Caucasian embryo but were later contaminated with HeLa cells. Thus, INT 407 cells are considered to be a HeLa derivative. Caco-2 cells were originally derived from a human colon carcinoma. INT 407 cells grow rapidly and are easy to maintain and manipulate in culture, while Caco-2 cells grow slower and are less tractable for some experimental manipulations. *Campylobacter* researchers have extensively used both INT 407 and Caco-2 cells over the past two decades as cellular model systems to dissect bacteria-host cell interactions. The main advantage of the *in vitro* cell culture model system utilized herein resides in the ability to perform several key experiments to assess the pathogenic behavior of *C. jejuni* with each type of epithelial cell. These experiments defined the kinetics of *C. jejuni-*host cell invasion and verified that active protein synthesis is required for *C. jejuni* to maximally invade both INT 407 and Caco-2 cells. Relevant to the experimental approach taken herein is that the synthetic profile of the bacteria present in the supernatant (i.e., medium overlying the epithelial cell monolayers) is similar to that observed for cell-associated bacteria, indicating that either transient association of the bacteria with the epithelial cells or exposure to the culture environment is sufficient to constitutively induce the altered synthetic response (Konkel and Cieplak, 1992). After defining these parameters, proteomic and transcriptomic approaches were used to identify putative virulence genes.

The proteomic (LC-MS/MS) and transcriptomic (RNA-Seq) analysis of *C. jejuni* with the INT 407 and Caco-2 cells was highly reproducible and there was a strong correlation between the two data sets. This correlation provided confidence that the appropriate methodologies were applied to dissect the bacterium’s adaptation to co-culture with host cells. Analysis of the data sets divided by cell type further revealed that *C. jejuni* responded to co-culture with the INT 407 in a similar manner to co-culture with Caco-2 cells. Relevant to this observation were the results of the phenotypic assays, where the temporal kinetics of *C. jejuni* internalization in INT 407 cells and Caco-2 cells were found to be indistinguishable. This observation demonstrates that *C. jejuni*, at least upon first interaction, does not distinguish between distinct cell lines with different cellular markers. Ultimately, kinetic RNA-Seq analysis allowed for global detection of transcripts, including those in low abundance, to identify genes whose expression increased and decreased from 2.5 to 4 h with both INT 407 and Caco-2 cells. This analysis led to the comprehensive lists of genes that are differentially expressed by *C. jejuni* in response to co-culture with two different human cell lines. This enabled identification of *C. jejuni* genes and gene products that are clearly important for the interaction of host cells.

The gene expression data provide insight into the environment encountered and metabolism of *C. jejuni* at a time when the bacteria are invading the epithelial cells. The growth conditions were clearly distinct from conditions at time 0, as evident by the number of upregulated (*n* = 23) and downregulated (*n* = 4) genes that belong to COG categories: ‘inorganic ion transport and metabolism,’ ‘amino acid transport and metabolism,’ and ‘posttranslational modification.’ Additionally, the downregulation of 70 genes associated with energy production and conversion (COG C) suggests that the bacteria are not rapidly growing during this time (Supplementary Table S2). Several of the genes upregulated during co-culture further suggest the environment has certain nutrients that are limiting, and that reactive oxygen/nitrogen species could be present. An increase was also observed in the expression of components that make up several iron acquisition systems, including transporters for ferri-transferins (e.g., *Cj0175c* and *Cj0176c*), ferri-enterochelin (e.g., *ceuD*), ferri-rhodotorulic acid (e.g., *Cj1658*), and hemin (e.g., *chuA* and *chuD*). While *C. jejuni* has an additional system to acquire soluble Fe^2+^ (Miller et al., 2009), the expression of the genes that comprise this system (e.g., *feoAB*) did not increase from 2.5 to 4 h. We also observed the upregulation of genes that encode proteins involved in mitigating oxygen/nitrogen species (e.g., *ctb, katA, perR, sodB*, and *ahpC*). It has been demonstrated in previous studies that the regulation of ion uptake and reactive oxygen/nitrogen detoxification is coordinated together by the Fur and PerR regulators (Butcher et al., 2015). Consistent with the increased protein synthesis and metabolic activity, the expression of 30 genes increased from 2.5 to 4 h. These genes belonged to the categories of ‘amino acid transport and metabolism,’ and ‘posttranslational modification.’ The importance of this observation is that *C. jejuni* is an asaccharolytic organism, meaning that it utilizes amino acids as its primary energy source for growth. The top four preferentially used amino acids are serine, aspartate, glutamate and proline (Leach et al., 1997). Specifically, transporters and enzymes that utilize each of the four amino acids serine (*sdaC*), aspartate and glutamate [*Cj0919c, Cj0920c, Cj0921c* (Peb1A), and *Cj0922c* (PebC)] (Stahl et al., 2012) and proline (*putA* and *putP*) were seen to increase from 2.4 to 4 h (Hofreuter et al., 2012). Furthermore, *ggt*, which encodes the enzyme γ-glutamyltranspeptidase that catalyzes the hydrolysis of glutamine and glutathione to glutamate, was also increased (Thompson and Gaynor, 2008). While a deficiency in amino acid uptake is dispensable for survival in rich medium, it appears to be critical for *in vivo* colonization (Velayudhan et al., 2004). Also observed was an increase in proteins that assist with oxidative protein folding, including a *dsbA* homolog (Grabowska et al., 2014). It appears that phosphate is not limiting in this environment, based on the observation that the expression of the PhosS/PhosR regulon did not change (Wosten et al., 2006). In summary, we propose that the increase of genes related to amino acid uptake and posttranslational modification supports the increased metabolic rate observed in the presence of cells.

The transcriptomic data suggest that *C. jejuni* senses factors upon co-culture with epithelial cells that mimic an environment with oxygen limitation. The RacRS two-component regulatory system is responsive to limited oxygen when alternative electron acceptors are present, controlling the production of fumarate from aspartate, as well as its transport and reduction to succinate (van der Stel et al., 2015). Of the 11 RacRS activated genes previously identified by microarray analysis (van der Stel et al., 2015), eight genes were upregulated by *C. jejuni* when co-cultured with epithelial cells (green highlighted genes in Supplementary Table S2). Conversely, there were several genes that are repressed by RacR, including *Cj0358* and genes among the three putative operons *aspA*-*dcuA, mfrXABE* (formerly annotated as *sdhABC*, Guccione et al., 2010), and *Cj0448c-Cj0449c* (red-highlighted genes in Supplementary Table S2). We propose that there are additional molecules or signals present in this environment that the *C. jejuni* senses to prepare for metabolism in an oxygen limited environment. This further supports the usefulness of the *in vitro* model and the data presented in this study.

Among the genes that were upregulated in the presence of host cells from 2.5 to 4 h are genes involved in chemotaxis and in the regulation of flagellar assembly. We selected *Cj0448c, cheBR, cetAB, flhF*, and *flgL* to generate gene deletions to perform phenotypic characterization. *Cj0448c, cheBR*, and *cetAB* are proposed to encode chemotaxis-related proteins, *flhF* is proposed to be a flagellar biosynthesis regulator protein (Elliott et al., 2009; Kanungpean et al., 2011; Chandrashekhar et al., 2015), and *flgL* is the flagellar hook junction protein. The motility, filament length, and invasion potential of all of the *C. jejuni* deletion mutants were then assessed. Part of the reason for generating mutations in these genes, and especially in *cetAB*, is because discrepancies have been reported in the phenotypic properties of these mutants (Hendrixson and DiRita, 2004; Elliott et al., 2009). Moreover, we felt it necessary to compare the phenotypes of these mutants in a single genetic background to determine the relative importance of each protein in *C. jejuni* cell invasion. These studies revealed that the filament length in the *Cj0448c, cheBR*, and *cetAB* deletion mutants was indistinguishable from that of the wild-type strain, whereas more than 95% of the *flhF* gene deletion mutants were lacking an observable filament. Consistent with the published literature, a decrease was observed in the zone of motility with the *cheBR* and *cetAB* deletion mutants on the MH agar plates. The invasion assays provided the most insight in the contribution of each system in bacteria–host interactions. None of the chemotaxis mutants (*Cj0448c, cheBR, cetAB*) demonstrated any defect in host cell invasion. This finding was in contrast to a previous study where a decrease was reported in the invasion potential (I/A times 100) for the *cetAB* deletion mutant (Elliott and Dirita, 2008). Not clear from the previously published work was whether the invasion deficiency of the *cetAB* mutant was statistically significant, as statistics were not applied. Nor was it clear whether the adherence potential of the *cetAB* mutant contributed to the decrease in invasion potential [(the number of bacterial invaded divided by the number of adherent bacteria) × 100], as the number of adherent bacteria was not reported. To ensure the reproducibility of the results presented herein, we generated *cetAB* deletion mutants independent of one another (separate electroporations) and found that all isolates demonstrated indistinguishable phenotypes. In contrast to the *Cj0448c, cheBR, cetAB* deletion mutants, the *flhF* and *flgL* mutants were grossly impaired in cell adherence and cell invasion. Complementation of the *flhF* and *flgL* deletion mutants *in cis* restored the invasive phenotype. Although *flhF* is predicted to be in a multigene operon, others have shown that an insertion of an antibiotic resistance gene in *flhF* does not have a polar effect (Liang and Connerton, 2018; Ren et al., 2018). Noteworthy is that the invasiveness of the *flhF* and *flgL* deletion mutants were indistinguishable from the *flaAB* deletion mutant. In addition, the I/A ratio of the 81–176 *flaAB* mutant was similar to a *C. jejuni* strain 81116 *flaAB* deletion mutant (Konkel et al., 2004). This work provides clarity of the role of *cetAB* in the process of *C. jejuni*-cell invasion. Moreover, the data herein demonstrates that: (1) chemotaxis proteins do not directly facilitate (and are not required for) host cell invasion; (2) the zone of motility, as evidenced by the migration of bacteria from the place at which they were spotted on a plate, does not correlate with host cell invasion potential.

*Campylobacter jejuni* genes of known and putative function that were upregulated over the course of the infection included *ciaB, katA, peb3A*, and *Cj1349c*. CiaB is required for flagellar dependent secretion of *Campylobacter* invasion antigens and is upregulated by *C. jejuni* cultured in the presence of a physiological concentration of the bile acid deoxycholate (Rivera-Amill et al., 2001; Malik-Kale et al., 2008), KatA detoxifies hydrogen peroxide and contributes to the colonization of chickens (Bingham-Ramos and Hendrixson, 2008), and Peb3 is a highly immunogenic N-glycosylated surface protein that may play a role in cell adhesion and 3-phosphoglycerate transport (Rangarajan et al., 2007; Shoaf-Sweeney et al., 2008; Min et al., 2009). One upregulated gene that encodes a protein that merits further study is *Cj1349c*. Interesting properties of the *Cj1349c* gene/protein included the following: (1) the gene has been implicated in contributing to infection upon screening a *C. jejuni* Tn mutant library in the gnotobiotic piglet model of campylobacteriosis (de Vries et al., 2017); and 2) the deduced amino acid sequence harbors putative fibronectin and fibrinogen binding protein domains. Studies are in progress to dissect the function of *Cj1349c*. Another upregulated gene of interest is *ppk* (aka *Cj1359* that encodes PPK1). Pina-Mimbela et al. (2015) found that polyphosphate kinase 1 and 2 (PPK1 and PPK2) mutants demonstrate altered outer membrane constituents and are reduced in the invasion of INT 407 compared to a *C. jejuni* wild-type strain. Moreover, 27 genes were observed to be upregulated and 12 genes were observed to be downregulated that belonged to the ‘uncategorized’ and ‘function unknown’ COG categories. Given that several of these *C. jejuni* genes have been implicated in colonization and/or disease [e.g., *Cj0448c* (Woodall et al., 2005), *Cj0511* (Karlyshev et al., 2014), *Cj1349c* (de Vries et al., 2017), *ciaB* (Rivera-Amill et al., 2001; Raphael et al., 2005; Malik-Kale et al., 2008; Naz, 2014), *mapA* (Shoaf-Sweeney et al., 2008; Johnson et al., 2014), *ppk* (Pina-Mimbela et al., 2015), and *peb3* (Rangarajan et al., 2007; Shoaf-Sweeney et al., 2008; Min et al., 2009)], future studies are warranted to dissect the function of the upregulated and downregulated genes that have previously not been categorized.

In summary, we have analyzed metabolic activity and used proteomic and transcriptomic approaches to investigate the response of a *C. jejuni* clinical strain to human INT 407 cells and human colonic Caco-2 cells. There was good correlation between the proteomic and transcriptomic data sets, providing confidence in the validity of the changes seen in the proteomic and transcriptomic data regarding *C. jejuni-*host cell interactions. Also striking was the finding that *C. jejuni* responds to INT 407 and Caco-2 cells in a similar fashion at both the cellular and molecular levels. The combined approach used in this study led to the identification of interesting genes/proteins whose expression and abundance changes in response to co-cultivation with human epithelial cells. Several observations demonstrated that *C. jejuni* rapidly adapted to co-culture with host cells, likely conferring a growth and/or survival advantage in this particular microenvironment. These observations included: (1) Co-culture of *C. jejuni* with epithelial cells stimulated the bacterial metabolic activity; (2) the number of internalized (gentamicin-protected) *C. jejuni* increased about two orders of magnitude during the interval from 30 to 180 min; (3) *C. jejuni* express gene products to acquire iron and amino acids and produce products to resist the toxic effects of reactive oxygen/nitrogen species; and (4) *C. jejuni* express gene products that specifically facilitate host cell interactions. Further characterization of the genes whose expression increases and decreases upon *C. jejuni* co-culture with epithelial cells will provide new insights into the infection process.

## Author Contributions

NN, GC, CG, JA, CP, and MK conceived and designed the experiments and analyzed the data. NN, GC, PT, CG, SH, CC, and MK performed the experiments. All authors wrote and reviewed the manuscript.

## Conflict of Interest Statement

The authors declare that the research was conducted in the absence of any commercial or financial relationships that could be construed as a potential conflict of interest.

## Footnotes

1 microbesonline.org

## References

[B1] Backert S. Hofreuter D. (2013). Molecular methods to investigate adhesion, transmigration, invasion and intracellular survival of the foodborne pathogen *Campylobacter jejuni*. *J. Microbiol. Methods* 95 8–23. 10.1016/j.mimet.2013.06.031 23872466

[B2] Bingham-Ramos L. K. Hendrixson D. R. (2008). Characterization of two putative cytochrome c peroxidases of *Campylobacter jejuni* involved in promoting commensal colonization of poultry. *Infect. Immun.* 76 1105–1114. 10.1128/IAI.01430-07 18086814 PMC2258805

[B3] Butcher J. Handley R. A. Van Vliet A. H. Stintzi A. (2015). Refined analysis of the *Campylobacter jejuni* iron-dependent/independent Fur- and PerR-transcriptomes. *BMC Genomics* 16:498. 10.1186/s12864-015-1661-7 26141822 PMC4491227

[B4] Candon H. L. Allan B. J. Fraley C. D. Gaynor E. C. (2007). Polyphosphate kinase 1 is a pathogenesis determinant in *Campylobacter jejuni*. *J. Bacteriol.* 189 8099–8108. 10.1128/JB.01037-07 17827292 PMC2168705

[B5] Chandrashekhar K. Gangaiah D. Pina-Mimbela R. Kassem I. I. Jeon B. H. Rajashekara G. (2015). Transducer like proteins of *Campylobacter jejuni* 81-176: role in chemotaxis and colonization of the chicken gastrointestinal tract. *Front. Cell Infect. Microbiol.* 5:46. 10.3389/fcimb.2015.00046 26075188 PMC4444964

[B6] Christensen J. E. Pacheco S. A. Konkel M. E. (2009). Identification of a *Campylobacter jejuni*-secreted protein required for maximal invasion of host cells. *Mol. Microbiol.* 73 650–662. 10.1111/j.1365-2958.2009.06797.x 19627497 PMC2764114

[B7] Cox J. Hein M. Y. Luber C. A. Paron I. Nagaraj N. Mann M. (2014). Accurate proteome-wide label-free quantification by delayed normalization and maximal peptide ratio extraction, termed MaxLFQ. *Mol. Cell Proteomics* 13 2513–2526. 10.1074/mcp.M113.031591 24942700 PMC4159666

[B8] de Vries S. P. Linn A. Macleod K. Maccallum A. Hardy S. P. Douce G. (2017). Analysis of *Campylobacter jejuni* infection in the gnotobiotic piglet and genome-wide identification of bacterial factors required for infection. *Sci. Rep.* 7:44283. 10.1038/srep44283 28281647 PMC5345035

[B9] Elliott K. T. Dirita V. J. (2008). Characterization of CetA and CetB, a bipartite energy taxis system in *Campylobacter jejuni*. *Mol. Microbiol.* 69 1091–1103. 10.1111/j.1365-2958.2008.06357.x 18631239 PMC2628428

[B10] Elliott K. T. Zhulin I. B. Stuckey J. A. Dirita V. J. (2009). Conserved residues in the HAMP domain define a new family of proposed bipartite energy taxis receptors. *J. Bacteriol.* 191 375–387. 10.1128/JB.00578-08 18952801 PMC2612422

[B11] Elmi A. Nasher F. Jagatia H. Gundogdu O. Bajaj-Elliott M. Wren B. (2016). *Campylobacter jejuni* outer membrane vesicle-associated proteolytic activity promotes bacterial invasion by mediating cleavage of intestinal epithelial cell E-cadherin and occludin. *Cell Microbiol.* 18 561–572. 10.1111/cmi.12534 26451973

[B12] Eucker T. P. Konkel M. E. (2012). The cooperative action of bacterial fibronectin-binding proteins and secreted proteins promote maximal *Campylobacter jejuni* invasion of host cells by stimulating membrane ruffling. *Cell Microbiol.* 14 226–238. 10.1111/j.1462-5822.2011.01714.x 21999233 PMC3262099

[B13] Eucker T. P. Samuelson D. R. Hunzicker-Dunn M. Konkel M. E. (2014). The focal complex of epithelial cells provides a signalling platform for interleukin-8 induction in response to bacterial pathogens. *Cell Microbiol.* 16 1441–1455. 10.1111/cmi.12305 24779413 PMC4146656

[B14] Everest P. H. Goossens H. Butzler J. P. Lloyd D. Knutton S. Ketley J. M. (1992). Differentiated Caco-2 cells as a model for enteric invasion by *Campylobacter jejuni* and *C. coli*. *J. Med. Microbiol.* 37 319–325. 10.1099/00222615-37-5-319 1433253

[B15] Flanagan R. C. Neal-Mckinney J. M. Dhillon A. S. Miller W. G. Konkel M. E. (2009). Examination of *Campylobacter jejuni* putative adhesins leads to the identification of a new protein, designated FlpA, required for chicken colonization. *Infect. Immun.* 77 2399–2407. 10.1128/IAI.01266-08 19349427 PMC2687328

[B16] Friis L. M. Pin C. Pearson B. M. Wells J. M. (2005). *In vitro* cell culture methods for investigating *Campylobacter invasion* mechanisms. *J. Microbiol. Methods* 61 145–160. 10.1016/j.mimet.2004.12.003 15722140

[B17] Galperin M. Y. Makarova K. S. Wolf Y. I. Koonin E. V. (2015). Expanded microbial genome coverage and improved protein family annotation in the COG database. *Nucleic Acids Res.* 43 D261–D269. 10.1093/nar/gku1223 25428365 PMC4383993

[B18] Gourley C. R. Negretti N. M. Konkel M. E. (2017). The food-borne pathogen *Campylobacter jejuni* depends on the AddAB DNA repair system to defend against bile in the intestinal environment. *Sci. Rep.* 7:14777. 10.1038/s41598-017-14646-9 29089630 PMC5665897

[B19] Grabowska A. D. Wywial E. Dunin-Horkawicz S. Lasica A. M. Wosten M. M. Nagy-Staron A. (2014). Functional and bioinformatics analysis of two *Campylobacter jejuni* homologs of the thiol-disulfide oxidoreductase, DsbA. *PLoS One* 9:e106247. 10.1371/journal.pone.0106247 25181355 PMC4152235

[B20] Guccione E. Hitchcock A. Hall S. J. Mulholland F. Shearer N. Van Vliet A. H. (2010). Reduction of fumarate, mesaconate and crotonate by Mfr, a novel oxygen-regulated periplasmic reductase in *Campylobacter jejuni*. *Environ. Microbiol.* 12 576–591. 10.1111/j.1462-2920.2009.02096.x 19919540

[B21] Hendrixson D. R. Akerley B. J. Dirita V. J. (2001). Transposon mutagenesis of *Campylobacter jejuni* identifies a bipartite energy taxis system required for motility. *Mol. Microbiol.* 40 214–224. 10.1046/j.1365-2958.2001.02376.x 11298288

[B22] Hendrixson D. R. DiRita V. J. (2004). Identification of *Campylobacter jejuni* genes involved in commensal colonization of the chick gastrointestinal tract. *Mol. Microbiol.* 52 471–484. 10.1111/j.1365-2958.2004.03988.x 15066034

[B23] Hofreuter D. Mohr J. Wensel O. Rademacher S. Schreiber K. Schomburg D. (2012). Contribution of amino acid catabolism to the tissue specific persistence of *Campylobacter jejuni* in a murine colonization model. *PLoS One* 7:e50699. 10.1371/journal.pone.0050699 23226358 PMC3511319

[B24] Johanesen P. A. Dwinell M. B. (2006). Flagellin-independent regulation of chemokine host defense in *Campylobacter jejuni*-infected intestinal epithelium. *Infect. Immun.* 74 3437–3447. 10.1128/IAI.01740-05 16714574 PMC1479283

[B25] Johnson J. G. Livny J. Dirita V. J. (2014). High-throughput sequencing of *Campylobacter jejuni* insertion mutant libraries reveals *mapA* as a fitness factor for chicken colonization. *J. Bacteriol.* 196 1958–1967. 10.1128/JB.01395-13 24633877 PMC4010991

[B26] Kanungpean D. Kakuda T. Takai S. (2011). Participation of CheR and CheB in the chemosensory response of *Campylobacter jejuni*. *Microbiology* 157 1279–1289. 10.1099/mic.0.047399-0 21292743

[B27] Karlyshev A. V. Thacker G. Jones M. A. Clements M. O. Wren B. W. (2014). *Campylobacter jejuni* gene *cj0511* encodes a serine peptidase essential for colonisation. *FEBS Open Bio.* 4 468–472. 10.1016/j.fob.2014.04.012 24918062 PMC4050187

[B28] Konkel M. E. Christensen J. E. Dhillon A. S. Lane A. B. Hare-Sanford R. Schaberg D. M. (2007). *Campylobacter jejuni* strains compete for colonization in broiler chicks. *Appl. Environ. Microbiol.* 73 2297–2305. 10.1128/AEM.02193-06 17293510 PMC1855682

[B29] Konkel M. E. Cieplak W. Jr. (1992). Altered synthetic response of *Campylobacter jejuni* to cocultivation with human epithelial cells is associated with enhanced internalization. *Infect. Immun.* 60 4945–4949. 1399005 10.1128/iai.60.11.4945-4949.1992 PMC258252

[B30] Konkel M. E. Garvis S. G. Tipton S. L. Anderson D. E. Jr. Cieplak W. Jr. (1997). Identification and molecular cloning of a gene encoding a fibronectin-binding protein (CadF) from *Campylobacter jejuni*. *Mol. Microbiol.* 24 953–963. 10.1046/j.1365-2958.1997.4031771.x 9220003

[B31] Konkel M. E. Klena J. D. Rivera-Amill V. Monteville M. R. Biswas D. Raphael B. (2004). Secretion of virulence proteins from *Campylobacter jejuni* is dependent on a functional flagellar export apparatus. *J. Bacteriol.* 186 3296–3303. 10.1128/JB.186.11.3296-3303.2004 15150214 PMC415756

[B32] Konkel M. E. Larson C. L. Flanagan R. C. (2010). *Campylobacter jejuni* FlpA binds fibronectin and is required for maximal host cell adherence. *J. Bacteriol.* 192 68–76. 10.1128/JB.00969-09 19880595 PMC2798237

[B33] Konkel M. E. Mead D. J. Cieplak W. Jr. (1993). Kinetic and antigenic characterization of altered protein synthesis by *Campylobacter jejuni* during cultivation with human epithelial cells. *J. Infect. Dis.* 168 948–954. 10.1093/infdis/168.4.948 8376841

[B34] Korlath J. A. Osterholm M. T. Judy L. A. Forfang J. C. Robinson R. A. (1985). A point-source outbreak of campylobacteriosis associated with consumption of raw milk. *J. Infect. Dis.* 152 592–596. 10.1093/infdis/152.3.592 4031557

[B35] Larson C. L. Samuelson D. R. Eucker T. P. O’loughlin J. L. Konkel M. E. (2013). The fibronectin-binding motif within FlpA facilitates *Campylobacter jejuni* adherence to host cell and activation of host cell signaling. *Emerg Microbes Infect* 2 e65. 10.1038/emi.2013.65 26038437 PMC3826066

[B36] Le S. Josse J. Husson F. (2008). FactoMineR: an R package for multivariate analysis. *J. Stat. Softw.* 25 1–18. 10.18637/jss.v025.i01

[B37] Leach S. Harvey P. Wali R. (1997). Changes with growth rate in the membrane lipid composition of and amino acid utilization by continuous cultures of *Campylobacter jejuni*. *J. Appl. Microbiol.* 82 631–640. 10.1111/j.1365-2672.1997.tb02873.x 9172406

[B38] Liang L. Connerton I. F. (2018). FlhF(T368A) modulates motility in the bacteriophage carrier state of *Campylobacter jejuni*. *Mol. Microbiol.* 110 616–633. 10.1111/mmi.14120 30230632 PMC6282759

[B39] Malik-Kale P. Parker C. T. Konkel M. E. (2008). Culture of *Campylobacter jejuni* with sodium deoxycholate induces virulence gene expression. *J. Bacteriol.* 190 2286–2297. 10.1128/JB.01736-07 18223090 PMC2293209

[B40] McSweegan E. Walker R. I. (1986). Identification and characterization of two *Campylobacter jejuni* adhesins for cellular and mucous substrates. *Infect. Immun.* 53 141–148. 2873103 10.1128/iai.53.1.141-148.1986 PMC260088

[B41] Mendiburu F. Simon R. (2015). Agricolae - Ten years of an open source statistical tool for experiments in breeding, agriculture and biology. *PeerJ PrePrints* 3:e1748v1 10.7287/peerj.preprints.1404v1

[B42] Miller C. E. Williams P. H. Ketley J. M. (2009). Pumping iron: mechanisms for iron uptake by *Campylobacter*. *Microbiology* 155 3157–3165. 10.1099/mic.0.032425-0 19696110

[B43] Min T. Vedadi M. Watson D. C. Wasney G. A. Munger C. Cygler M. (2009). Specificity of *Campylobacter jejuni* adhesin PEB3 for phosphates and structural differences among its ligand complexes. *Biochemistry* 48 3057–3067. 10.1021/bi802195d 19236052

[B44] Naz N. (2014). *Reinvestigation into the Mechanisms of Campylobacter jejuni Invasion of Intestinal Epithelial Cells.* Doctoral Dessertation, London School of Hygiene & Tropical Medicine, London.

[B45] Neal-McKinney J. M. Konkel M. E. (2012). The *Campylobacter jejuni* CiaC virulence protein is secreted from the flagellum and delivered to the cytosol of host cells. *Front. Cell Infect. Microbiol.* 2:31. 10.3389/fcimb.2012.00031 22919623 PMC3417660

[B46] Negretti N. M. Gourley C. R. Clair G. Adkins J. N. Konkel M. E. (2017). The food-borne pathogen *Campylobacter jejuni* responds to the bile salt deoxycholate with countermeasures to reactive oxygen species. *Sci. Rep.* 7:15455. 10.1038/s41598-017-15379-5 29133896 PMC5684402

[B47] Palyada K. Sun Y. Q. Flint A. Butcher J. Naikare H. Stintzi A. (2009). Characterization of the oxidative stress stimulon and PerR regulon of *Campylobacter jejuni*. *BMC Genomics* 10:481. 10.1186/1471-2164-10-481 19835633 PMC2772861

[B48] Panigrahi P. Losonsky G. Detolla L. J. Morris J. G. Jr. (1992). Human immune response to *Campylobacter jejuni* proteins expressed *in vivo*. *Infect. Immun.* 60 4938–4944. 1399004 10.1128/iai.60.11.4938-4944.1992 PMC258251

[B49] Pina-Mimbela R. Madrid J. A. Kumar A. Torrelles J. B. Rajashekara G. (2015). Polyphosphate kinases modulate *Campylobacter jejuni* outer membrane constituents and alter its capacity to invade and survive in intestinal epithelial cells *in vitro*. *Emerg Microbes Infect* 4 e77. 10.1038/emi.2015.77 26714783 PMC4715166

[B50] Rangarajan E. S. Bhatia S. Watson D. C. Munger C. Cygler M. Matte A. (2007). Structural context for protein N-glycosylation in bacteria: The structure of PEB3, an adhesin from *Campylobacter jejuni*. *Protein Sci.* 16 990–995. 10.1110/ps.062737507 17456748 PMC2206636

[B51] Raphael R. H. Monteville M. R. Klena J. D. Joens L. A. Konkel M. E. (2005). “Interactions of *Campylobacter jejuni* with non-professional phagocytic cells,” in *Campylobacter Molecular and Cellular Biology*, eds Ketley J. M. Konkel M. E. (Norfolk: Horizon Bioscience), 397–413.

[B52] Ren F. Lei T. Song Z. Yu T. Li Q. Huang J. (2018). Could FlhF be a key element that controls *Campylobacter jejuni* flagella biosynthesis in the initial assembly stage? *Microbiol. Res.* 207 240–248. 10.1016/j.micres.2017.12.006 29458860

[B53] Rey F. E. Faith J. J. Bain J. Muehlbauer M. J. Stevens R. D. Newgard C. B. (2010). Dissecting the *in vivo* metabolic potential of two human gut acetogens. *J. Biol. Chem.* 285 22082–22090. 10.1074/jbc.M110.117713 20444704 PMC2903421

[B54] Rivera-Amill V. Kim B. J. Seshu J. Konkel M. E. (2001). Secretion of the virulence-associated *Campylobacter* invasion antigens from *Campylobacter jejuni* requires a stimulatory signal. *J. Infect. Dis.* 183 1607–1616. 10.1086/320704 11343209

[B55] Ruiz-Palacios G. M. (2007). The health burden of *Campylobacter* infection and the impact of antimicrobial resistance: playing chicken. *Clin. Infect. Dis.* 44 701–703. 10.1086/509936 17278063

[B56] Shoaf-Sweeney K. D. Larson C. L. Tang X. Konkel M. E. (2008). Identification of *Campylobacter jejuni* proteins recognized by maternal antibodies of chickens. *Appl. Environ. Microbiol.* 74 6867–6875. 10.1128/AEM.01097-08 18805999 PMC2583476

[B57] Stahl M. Butcher J. Stintzi A. (2012). Nutrient acquisition and metabolism by *Campylobacter jejuni*. *Front. Cell Infect. Microbiol.* 2:5 10.3389/fcimb.2012.00005 PMC3417520 22919597

[B58] Sulaeman S. Hernould M. Schaumann A. Coquet L. Bolla J. M. De E. (2012). Enhanced adhesion of *Campylobacter jejuni* to abiotic surfaces is mediated by membrane proteins in oxygen-enriched conditions. *PLoS One* 7:e46402. 10.1371/journal.pone.0046402 23029510 PMC3460892

[B59] Thompson S. A. Gaynor E. C. (2008). *Campylobacter jejuni* host tissue tropism: a consequence of its low-carb lifestyle? *Cell Host Microbe* 4 409–410. 10.1016/j.chom.2008.10.010 18996338 PMC2762321

[B60] van der Stel A. X. Van Mourik A. Heijmen-Van Dijk L. Parker C. T. Kelly D. J. Van De Lest C. H. (2015). The *Campylobacter jejuni* RacRS system regulates fumarate utilization in a low oxygen environment. *Environ. Microbiol.* 17 1049–1064. 10.1111/1462-2920.12476 24707969

[B61] Velayudhan J. Jones M. A. Barrow P. A. Kelly D. J. (2004). L-serine catabolism via an oxygen-labile L-serine dehydratase is essential for colonization of the avian gut by *Campylobacter jejuni*. *Infect. Immun.* 72 260–268. 10.1128/IAI.72.1.260-268.2004 14688104 PMC343963

[B62] Vizcaino J. A. Csordas A. Del-Toro N. Dianes J. A. Griss J. Lavidas I. (2016). 2016 update of the PRIDE database and its related tools. *Nucleic Acids Res.* 44 D447–D456. 10.1093/nar/gkv1145 26527722 PMC4702828

[B63] Woodall C. A. Jones M. A. Barrow P. A. Hinds J. Marsden G. L. Kelly D. J. (2005). *Campylobacter jejuni* gene expression in the chick cecum: evidence for adaptation to a low-oxygen environment. *Infect. Immun.* 73 5278–5285. 10.1128/IAI.73.8.5278-5285.2005 16041056 PMC1201244

[B64] Wosten M. M. Parker C. T. Van Mourik A. Guilhabert M. R. Van Dijk L. Van Putten J. P. (2006). The *Campylobacter jejuni* PhosS/PhosR operon represents a non-classical phosphate-sensitive two-component system. *Mol. Microbiol.* 62 278–291. 10.1111/j.1365-2958.2006.05372.x 16956379

[B65] Yao R. Alm R. A. Trust T. J. Guerry P. (1993). Construction of new *Campylobacter* cloning vectors and a new mutational *cat* cassette. *Gene* 130 127–130. 10.1016/0378-1119(93)90355-7 8344519

