# Supplementary material for: Campylobacter jejuni Demonstrates Conserved Proteomic and Transcriptomic Responses When Co-cultured With Human INT 407 and Caco-2 Epithelial Cells
Source: Front Microbiol. 2019 Apr 11;10:755. doi: 10.3389/fmicb.2019.00755 (PMC6470190; doi:10.3389/fmicb.2019.00755)

***Campylobacter jejuni* demonstrates conserved proteomic and transcriptomic responses when co-cultured with human INT 407 and Caco-2 epithelial cells**

Nicholas M. Negretti,[1] Geremy Clair,[2] Prabhat K. Talukdar,[1] Christopher R. Gourley,[1] Steven Huynh,[3] Joshua N. Adkins,[2] Craig T. Parker,[3] Colby M. Corneau, [1] and Michael E. Konkel[1]*

[1] School of Molecular Biosciences, College of Veterinary Medicine, Washington State University, Pullman, WA, USA, 99164-7520

[2] Integrative Omics, Pacific Northwest National Laboratory, 902 Battelle Boulevard, Richland, Washington 99352

[3] Produce Safety and Microbiology, USDA Agricultural Research Service, 800 Buchanan St., Albany CA 94710

SUPPLEMENTAL FIGURES

Supplementary Figure 1. *C. jejuni* internalization of epithelial cells significantly increases with time. *C. jejuni* were incubated with INT 407 cells or Caco-2 cells for 1 hour, 2.5 hour, and 4 hour and the number of internalized bacteria was determined using *C. jejuni* strain 81-176 with INT 407 cells **(A)**, *C. jejuni* strain 81-176 with Caco-2 cells **(B)**, *C. jejuni* strain F38011 with INT 407 cells **(C)**, and *C. jejuni* strain F38011 with Caco-2 cells **(D)** by the gentamicin-protection assay. The number of gentamicin-protected bacteria/well of a 24-well tissue culture plate was determined by direct plate counts after lysing the monolayers with a solution of 0.1% Triton X-100. Values represent the percent relative invasion (number of gentamicin-protected bacteria/well of a 24-well tissue culture tray at 4 hour ± the standard deviations). Significant differences were determined by one-way ANOVA followed by Sidak's multiple comparisons test (* $p < 0.05$).

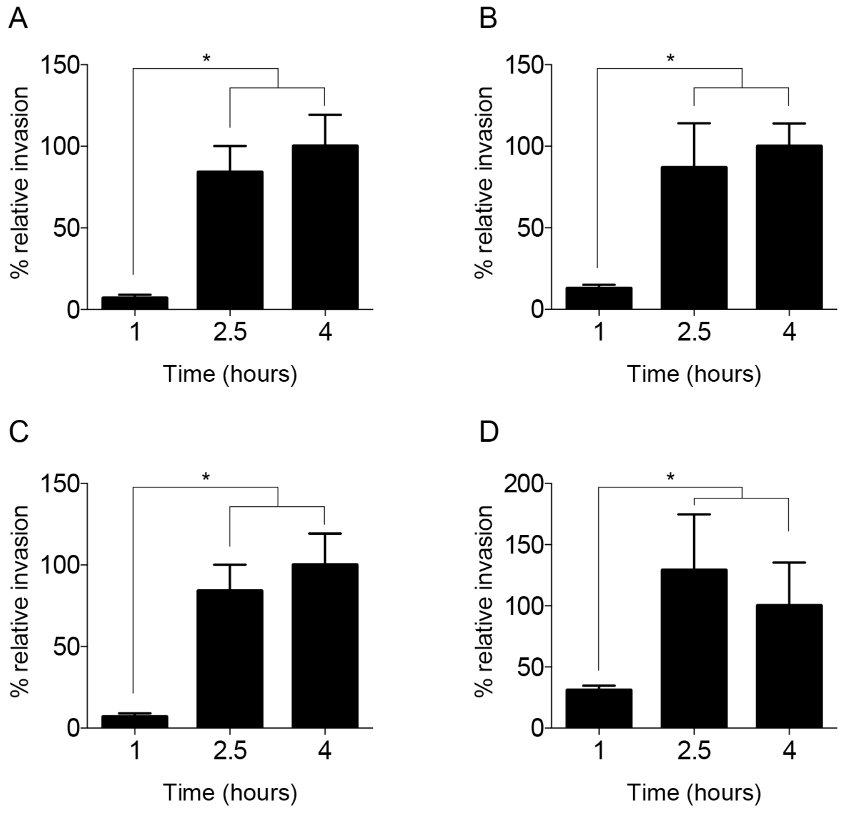

Supplementary Figure 2. Co-cultivation of *C. jejuni* strain 81-176 with viable INT 407 cells and Caco-2 epithelial cells alters protein profiles. Proteins that were significantly altered in abundance (p-value < 0.05 and $\log_2$ fold-change > 0.6) during co-cultivation with INT 407 and Caco-2 cells for 4 hours were compared. Protein abundance changes during co-incubation with host cells were compared to a baseline of culture in Mueller-Hinton broth for 4 hours.

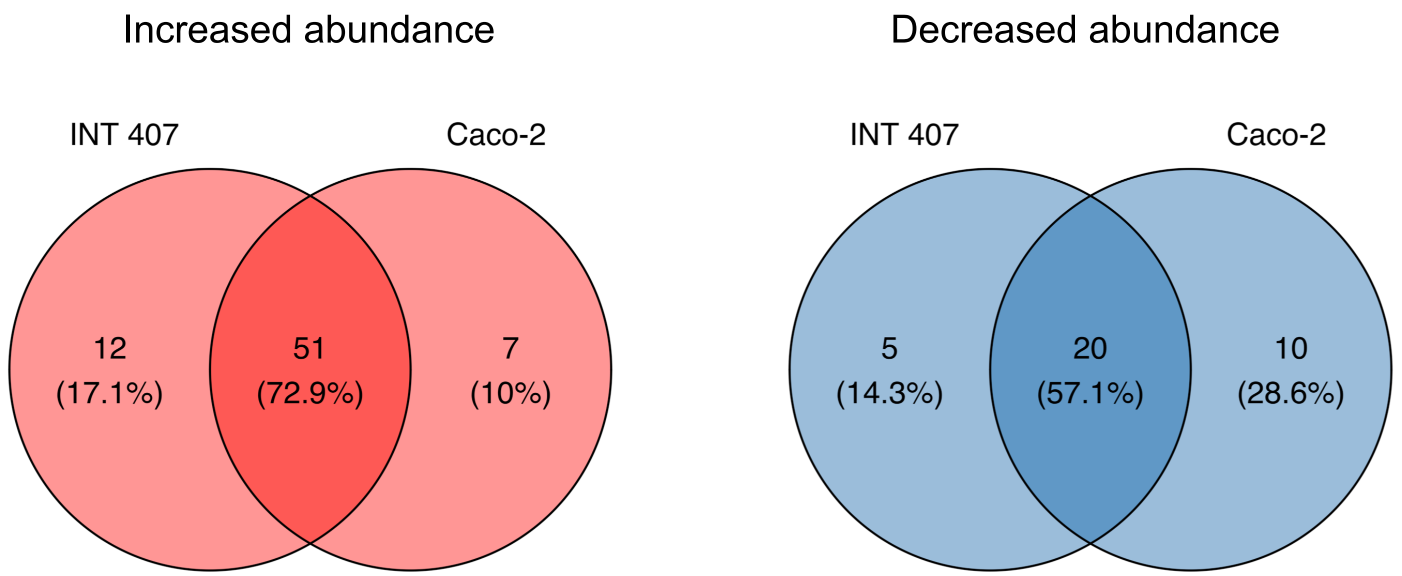

Supplementary Figure 3. Co-cultivation of *C. jejuni* strain 81-176 with viable INT 407 cells and Caco-2 epithelial cells alters gene expression. *C. jejuni* was co-cultured with INT 407 and Caco-2 cells for either 2.5 or 4 hours. Differentially expressed genes were compared for each cell type, at each time point to identify specific host-cell independent responses. Genes that were significantly upregulated or downregulated had a Benjamini-Hochberg adjusted p value of less than 0.1 ($q < 0.1$) as determined by a Wald test implemented in DESeq2.

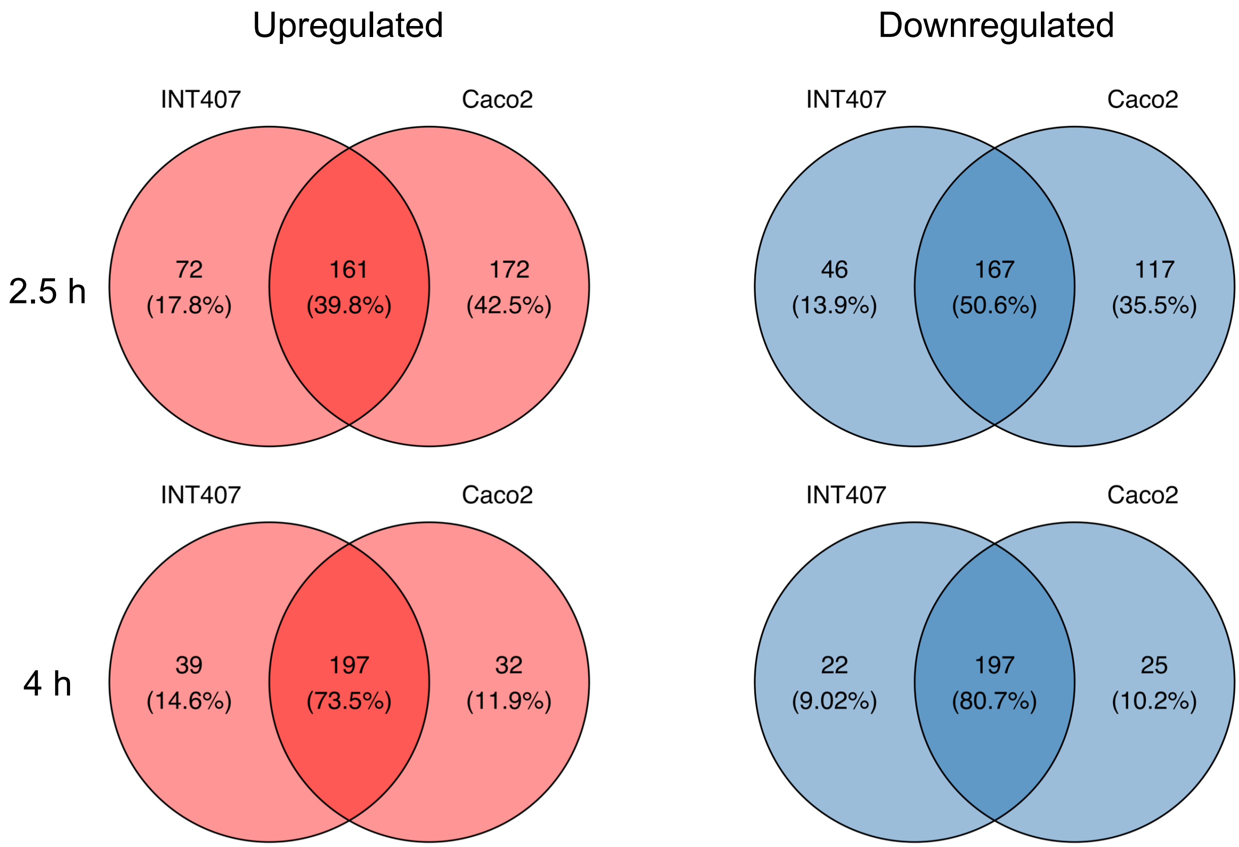

Supplementary Figure 4. Transcriptomic analyses of *C. jejuni* cultivated with epithelial cells unmask putative virulence genes. RNA-Seq experiments were performed using RNA extracted from *C. jejuni* strain 81-176 to identify genes whose expression changes in response to culture with INT 407 cells and/or Caco-2 cells. The bars above the 0 represent upregulated genes and the bars below 0 indicated downregulated genes. The checkered boxes represent the genes upregulated or downregulated at all of the time points tested. Genes that were significantly upregulated or downregulated had a Benjamini-Hochberg adjusted p value of less than 0.1 ($q < 0.1$).

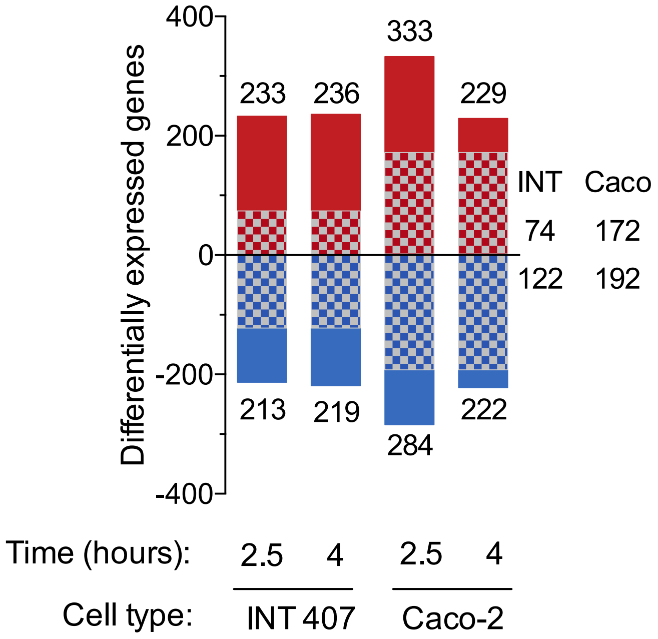

Supplement: Supplementary file 6 [file Data_Sheet_1.PDF]
